# Translatability Analysis of National Institutes of Health–Funded Biomedical Research That Applies Artificial Intelligence

**DOI:** 10.1001/jamanetworkopen.2021.44742

**Published:** 2022-01-24

**Authors:** Feyisope R. Eweje, Suzie Byun, Rajat Chandra, Fengling Hu, Ihab Kamel, Paul Zhang, Zhicheng Jiao, Harrison X. Bai

**Affiliations:** 1 Students, Perelman School of Medicine at University of Pennsylvania, Philadelphia; 2 Department of Radiology and Radiological Sciences, Johns Hopkins University, Baltimore, Maryland; 3 Department of Pathology and Laboratory Medicine, Hospital of the University of Pennsylvania, Philadelphia; 4 Department of Diagnostic Imaging, Rhode Island Hospital and Warren Alpert Medical School of Brown University, Providence, Rhode Island

## Abstract

**Importance:**

Despite the rapid growth of interest and diversity in applications of artificial intelligence (AI) to biomedical research, there are limited objective ways to characterize the potential for use of AI in clinical practice.

**Objective:**

To examine what types of medical AI have the greatest estimated translational impact (ie, ability to lead to development that has measurable value for human health) potential.

**Design, Setting, and Participants:**

In this cohort study, research grants related to AI awarded between January 1, 1985, and December 31, 2020, were identified from a National Institutes of Health (NIH) award database. The text content for each award was entered into a Natural Language Processing (NLP) clustering algorithm. An NIH database was also used to extract citation data, including the number of citations and approximate potential to translate (APT) score for published articles associated with the granted awards to create proxies for translatability.

**Exposures:**

Unsupervised assignment of AI-related research awards to application topics using NLP.

**Main Outcomes and Measures:**

Annualized citations per $1 million funding (ACOF) and average APT score for award-associated articles, grouped by application topic. The APT score is a machine-learning based metric created by the NIH Office of Portfolio Analysis that quantifies the likelihood of future citation by a clinical article.

**Results:**

A total of 16 629 NIH awards related to AI were included in the analysis, and 75 applications of AI were identified. Total annual funding for AI grew from $17.4 million in 1985 to $1.43 billion in 2020. By average APT, interpersonal communication technologies (0.488; 95% CI, 0.472-0.504) and population genetics (0.463; 95% CI, 0.453-0.472) had the highest translatability; environmental health (ACOF, 1038) and applications focused on the electronic health record (ACOF, 489) also had high translatability. The category of applications related to biochemical analysis was found to have low translatability by both metrics (average APT, 0.393; 95% CI, 0.388-0.398; ACOF, 246).

**Conclusions and Relevance:**

Based on this study's findings, data on grants from the NIH can apparently be used to identify and characterize medical applications of AI to understand changes in academic productivity, funding support, and potential for translational impact. This method may be extended to characterize other research domains.

## Key Points

**Question:**

Which applications of artificial intelligence (AI) in biomedical research could generate the most translational value (ie, ability to lead to development that has measurable value for human health)?

**Findings:**

In this cohort study using bibliometric analysis, 2 citation-based metrics were used as proxies for translational impact, and 75 unique medical applications of AI were identified from a set of 16 629 National Institutes of Health awards related to AI, using natural language processing. Varied applications were found to have high translatability, but bias against applications in basic biochemical analysis was evident.

**Meaning:**

These findings suggest that National Institutes of Health award data applications of AI in biomedical research are not equivalent: some demonstrate greater potential for translational impact than others.

## Teaser

This cohort study examines the translatability of findings from studies on artificial intelligence funded by the National Institutes of Health (NIH).

## Introduction

Artificial intelligence (AI) has the potential for transformational changes in health care. As early as the 1980s,^1^ it was understood that AI tools could eventually play a major role as expert consultants to physicians by using insights from data that may not be deemed actionable by human interpretation. From convolutional neural networks for imaging-based solid organ cancer screening^2,3,4^ to natural language processing (NLP) to estimate the probability of diagnoses with data from the electronic health record,^5,6,7^ the breadth of AI-powered technologies affecting our understanding of human health and health care delivery processes has rapidly expanded in recent years.^8^

Yet despite the exponential growth in academic research involving AI in medicine,^9^ it remains difficult to understand which applications have been associated with the greatest clinical impact and which applications have the greatest potential for future impact. Maximizing clinical translation (ie, ability to lead to development that has measurable value for human health) is a challenge for AI-powered biomedical research with many hurdles limiting innovations, including prospective studies, external cohort generalization, and difficulties integrating into existing clinical workflow.^10,11,12^ This problem has been described as the growing excitement around AI in health care despite limited examples of ways AI has tangibly changed clinical practice.^13,14^

A potential means of characterizing translation of AI is through research funding and related bibliometric data provided by the National Institutes of Health (NIH). As the world’s largest public funder of health research, the NIH has among its mandates to “expand the knowledge base in medical and associated sciences...and ensure continued high return on the public investment in research.”^15^ Studies have used NIH grant data to investigate academic productivity and translational value in biomedical research, from studying patent generation per unit of NIH funding to investigating the contribution of NIH funding in new drug approvals.^16,17^ Another study analyzed NIH funding for machine learning, but did not assess translational value.^18^ However, it has been reported that unsupervised NLP (ie, automatic generation of document categories without a priori knowledge) can segment NIH awards by topic similarity using text descriptions of the awards.^19^

By combining elements of each of these analyses, including translational value metrics, focus on AI-related NIH awards, and unsupervised NLP, it may be possible to quantifiably address the issue of which applications of AI have the greatest potential translational impact. In this cohort study, we strictly define the scope of AI applications in health care using unsupervised categorization of NIH awards and use bibliometric data provided by the NIH to investigate potential translational impact of various applications.

## Methods

### Data Collection

The NIH Research Portfolio Online Reporting Tools Expenditures and Results (RePORTER) search engine was queried for awards related to AI from January 1, 1985, to December 31, 2020, using a query of AI-related terms (eTable 1 and eMethods in the Supplement, defining artificial intelligence). Awards under activity codes T (training programs) and Z (intramural awards to NIH institutes) were excluded from the analysis because these awards often do not detail a focused area of proposed study. Subprojects, individual projects within multicomponent award applications, were also excluded from analysis. In addition, the RePORTER query returns a collection of academic articles that were produced in relation to the awards. These data include PubMed identification numbers, which were used to separately query the NIH iCite platform^20^ for citation information related to these articles. This cohort study involved nonhuman data and, per Common Rule 45 CFR 46.116(d)(4), was exempted from institutional review board review and the requirement for informed consent. This study followed the Strengthening the Reporting of Observational Studies in Epidemiology (STROBE) reporting guideline for cohort studies.

### Feature Extraction

eFigure 1 in the Supplement depicts the NLP pipeline. The title, abstract, and public health relevance statement from each award were combined as the input for text analysis. Stop words, which are common words with little semantic value (eg, the), were removed. Training features consisted of lemmatized unigrams (1-word sequences) and bigrams (2-word sequences) vectorized with term frequency-inverse document frequency (TF-IDF) weighting. Terms present across more than 10% of the document corpus were excluded from the feature set. The feature set was further narrowed to the 500 terms with the highest TF-IDF values summed across the corpus.

### Unsupervised Clustering

The topic identification algorithm was implemented with k-means clustering—an unsupervised machine learning algorithm that identifies clusters of related data points based on minimization of geometric distance between points assigned to a given cluster. As such, awards were assigned to the single topic that best characterized the text content of the award. The k-means algorithm was implemented with minibatches,^21^ in which multiple iterations with randomly selected partitions of the data set are conducted with each trial, with a batch size of 1024 and 100 iterations. The constant denoting of the number of clusters (K) was empirically determined by monitoring the silhouette score, a metric that reflects minimization of the mean intracluster distance and maximization of the mean nearest-cluster distance, with modulation of K (eFigure 2 in the Supplement). After K was selected, 50 training trials were conducted to create the final clusters, with the trial that maximized the silhouette coefficient chosen as the representative output.

### Cluster Validation

Each cluster created by the k-means algorithm was manually assigned a descriptive label based on the words selected as cluster-characteristic features by the algorithm and the content of award abstracts assigned to the cluster. Clusters with a silhouette score less than 0 (indicating poor assignment of the constituent awards) were excluded from further analysis. To validate the cluster topics, 2 of us (S.B. and R.C.) blinded to the awards’ cluster assignments manually assigned 200 randomly selected awards according to the k-means–defined topics. We also determined the fraction of awards in select k-means categories that were assigned to similarly defined research, condition, and disease categories, categories of research funding first generated by the NIH in 2008 (eMethods in the Supplement, cluster validation).

### Statistical Analysis

The overall award sample was characterized by comparing awards granted in 2008 and earlier with awards granted in 2009 and later, which is an inflection point corresponding with the passage of the Health Information Technology for Economic and Clinical Health (HITECH) act in the US.^22^ We applied log-likelihood ratios of document frequency to the TF-IDF feature set to determine the 10 most comparatively enriched terms within the 2 time periods.

Two metrics were used to quantify the likelihood of translational clinical impact based on articles associated with each NIH award. These metrics were applied to 3 collections of data: awards grouped by the funding NIH institute (eg, National Cancer Institute, National Institute on Aging), k-means–identified individual applications of AI, and applications grouped by general category. First, we calculated annualized citations per $1 million of funding (ACOF). For example, an article receiving 100 citations during 5 years received 20 annualized citations. Second, we calculated the average approximate potential to translate (APT) score for associated articles. The APT is a metric created by the NIH Office of Portfolio Analysis that has been demonstrated to be predictive of the likelihood of future citation by a clinical research article as an indicator of translation. Generated using a machine learning approach, the APT score is based on a data set of more than 9 million published biomedical research articles and outperformed academic experts in predicting clinical translation.^23^

We also sought to characterize funding growth for each identified application of AI. An exponential fit was made of each cluster’s annual funding over the study period to evaluate the estimated annual growth rate. In addition, to identify significant proportionality differences between general application categories by funding mechanism (eg, R01), the distributions of awards by funding mechanism were compared using exact binomial tests at significance level α = .05 with post hoc Bonferroni correction. All values with uncertainty are reported with a 95% CI. All analyses were conducted using Python, version 3.8 (Python Software Foundation). All code used to conduct analyses in this study is publicly available.

## Results

### Study Sample

Table 1 describes the sample of awards, characterized as pre-HITECH and post-HITECH. A total of 16 629 awards were identified for inclusion in the study. The awards totaled $7 177 080 553 in funding and had associated articles with an average APT of 0.422 (95% CI, 0.421-0.423) and ACOF of 301. The average APT significantly increased between the pre- and post-HITECH periods (0.390 vs 0.433; *P* < .001), but the ACOF decreased by 38% (from 444 to 275). The most enriched TF-IDF features by log-likelihood ratio included *lesion*, *physician*, and *interpretation* pre-HITECH and *ehr*, *big*, and *deep* post-HITECH. Overall funding grew from $17.4 million in 1985 to $1.43 billion in 2020 (eFigure 3 in the Supplement).

**Table 1.** National Institutes of Health–Funded Research Applying Artificial Intelligence for the HITECH Act

Variable	Pre-HITECH (2008 and earlier)	Post-HITECH (2009 and later)	Overall
No. of awards	1818	14 811	16 629
Total funding, $	1 090 391 998	6 086 688 555	7 177 080 553
Annualized citations per $1 million funding	444	275	301
Average approximate potential to translate (95% CI)	0.390 (0.388-0.393)^a^	0.433 (0.432-0.434)^a^	0.422 (0.421-0.423)^a^
Enriched features	base, prototype, artificial, lesion, physician, intelligence, mass, simulation, interpretation, procedure	ehr, big, deep, asd, youth, leverage, personalized, trajectory, autism, inform	NA

Abbreviations: HITECH, Health Information Technology for Economic and Clinical Health; NA, not applicable.

^a^ Significant at *P* < .001.

### Translatability by NIH Institute

**Figure 1.**
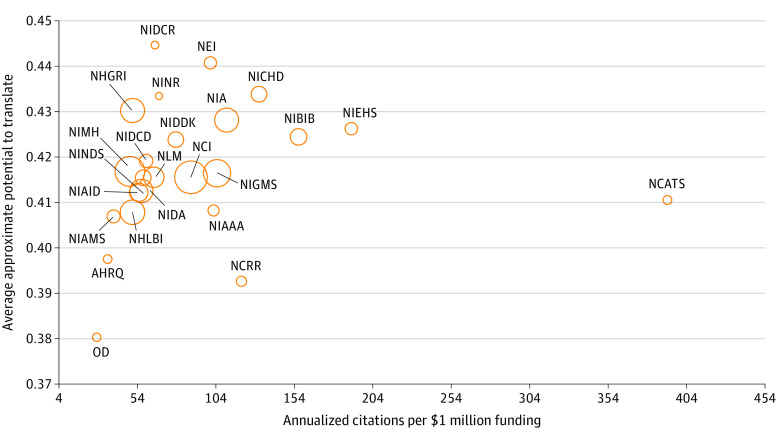
Translatability of National Institutes of Health–Funded Biomedical Research Applying Artificial Intelligence, by Institute The areas of the bubbles reflect the relative amount of research funding allocated by each National Institutes of Health institute. Only institutes that granted more than 100 awards during the study period are shown. AHRQ indicates Agency for Health Research and Quality; NCI, National Cancer Institute; NCRR, National Center for Research Resources; NEI, National Eye Institute; NHGRI, National Human Genome Research Institute; NHLBI, National Heart, Lung, and Blood Institute; NIA, National Institute on Aging; NIAAA, National Institute on Alcohol Abuse and Alcoholism; NIAID, National Institute of Allergy and Infectious Diseases; NIAMS, National Institute of Arthritis and Musculoskeletal and Skin Diseases; NIBIB, National Institute of Biomedical Imaging and Bioengineering; NICHD, Eunice Kennedy Shriver National Institute of Child Health and Human Development; NIDA, National Institute on Drug Abuse; NIDCD, National Institute on Deafness and Other Communication Disorders; NIDCR, National Institute of Dental and Craniofacial Research; NIDDK, National Institute of Diabetes and Digestive and Kidney Diseases; NIEHS, National Institute of Environmental Health Sciences; NIGMS, National Institute of General Medical Sciences; NIMH, National Institute of Mental Health; NINDS, National Institute of Neurological Disorders and Stroke; NINR, National Institute of Nursing Research; and NLM, National Library of Medicine.

The ACOF and average APT among NIH institutes are shown in Figure 1 and eTable 2 in the Supplement. Among institutes that granted more than 100 AI-related awards over the study period, the National Center for Advancing Translational Sciences funded the most translatable awards by ACOF (n, 392). Other high translatability institutes by ACOF included the National Institute of Environmental Health Sciences (ACOF, 190) and the National Institute of Biomedical Imaging and Bioengineering (ACOF, 157). The National Institute of Dental and Craniofacial Research (average APT, 0.445; 95% CI, 0.417-0.473) and National Eye Institute (average APT, 0.441; 95% CI, 0.426-0.455) produced the most translatable awards by average APT. The Agency for Healthcare Research and Quality and Office of the Director, institutes with research supportive missions, had the lowest translatability by both metrics.

### Applications of AI

Of the total 16 629 awards, 12 459 were sorted into 75 meaningfully descript applications of AI applications in biomedical research (Table 2); the remaining awards were assigned to clusters with silhouette scores less than 0 (ie, ill-defined topic assignments). The defined applications showed frequent overlap with the research, condition, and disease categories (eTable 3 in the Supplement) and fair to moderate agreement in award assignment with the manual raters (eTable 4 in the Supplement). Cluster-characteristic terms (eTable 5 in the Supplement) and a list of awards representative of each cluster (eTable 6 in the Supplement) were also described.

**Table 2.** National Institutes of Health–Funded Applications of Artificial Intelligence in Biomedical Research

Application	No. of granted awards	Total funding (1985-2020), $	Annualized citations per $1 million funding	Average APT (95% CI)	Estimated annual growth rate (95% CI)	Silhouette score
**Neurologic**						
Total	1552	812 472 532	222	0.426 (0.422-0.430)	0.457 (0.429-0.485)	
Alzheimer disease	321	241 056 913	158	0.421 (0.413-0.430)	0.482 (0.424-0.540)	0.255
Neural circuits	361	177 492 200	135	0.437 (0.427-0.447)	0.312 (0.265-0.359)	0.067
Other dementia	148	99 430 729	609	0.436 (0.429-0.443)	0.609 (0.512-0.705)	0.086
Stroke	176	88 644 658	194	0.377 (0.366-0.389)	0.508 (0.470-0.546)	0.279
Motor function	192	80 329 648	157	0.429 (0.415-0.443)	0.460 (0.335-0.585)	0.083
Memory	149	55 412 488	162	0.421 (0.405-0.437)	0.462 (0.387-0.537)	0.158
EEG	104	36 783 357	317	0.442 (0.424-0.459)	0.206 (0.155-0.256)	0.173
Sleep	101	33 322 539	211	0.447 (0.424-0.469)	0.869 (0.667-1.072)	0.372
**Genetics**						
Total	1632	732 428 485	352	0.428 (0.425-0.431)	0.157 (0.139-0.176)	
Regulatory genetics	280	134 538 181	198	0.429 (0.420-0.439)	0.193 (0.166-0.220)	0.062
Clinically significant genetic variation	236	117 453 892	327	0.442 (0.433-0.451)	0.203 (0.158-0.249)	0.153
Molecular genetics	246	105 378 437	421	0.418 (0.409-0.426)	0.103 (0.084-0.123)	0.100
Population genetics	130	76 684 671	563	0.463 (0.453-0.472)	0.056 (0.002-0.109)	0.153
Familial genetics	129	73 961 475	374	0.441 (0.430-0.453)	0.244 (0.144-0.344)	0.088
Gene mapping	166	61 918 552	485	0.399 (0.389-0.410)	0.303 (0.248-0.358)	0.052
Mouse modeling	167	60 846 677	207	0.401 (0.386-0.416)	0.155 (0.104-0.206)	0.087
Functional mutations	162	56 859 122	393	0.404 (0.394-0.415)	0.230 (0.181-0.278)	0.195
RNA analysis	116	44 787 478	287	0.439 (0.423-0.456)	0.179 (0.153-0.206)	0.228
**Mental health**						
Total	1361	648 405 425	269	0.426 (0.422-0.430)	0.337 (0.272-0.402)	
Pain	188	145 739 494	142	0.431 (0.418-0.443)	0.441 (0.173-0.709)	0.414
Autism spectrum disorder	192	97 331 715	243	0.458 (0.447-0.469)	0.230 (0.196-0.265)	0.236
Alcohol use	218	86 802 222	402	0.412 (0.403-0.422)	0.177 (0.144-0.209)	0.192
Other mental health	148	79 154 534	255	0.446 (0.433-0.459)	0.447 (0.384-0.509)	0.126
Adolescent psychiatry	155	63 322 086	275	0.387 (0.373-0.400)	0.233 (0.202-0.265)	0.100
Other child development	164	53 656 918	272	0.431 (0.417-0.445)	0.216 (0.190-0.243)	0.164
Depression	104	45 168 514	147	0.414 (0.391-0.438)	0.699 (0.498-0.900)	0.185
Suicidality	79	40 633 475	170	0.380 (0.362-0.398)	0.855 (0.632-1.079)	0.455
Schizophrenia	113	36 596 467	806	0.438 (0.427-0.448)	0.105 (0.044-0.166)	0.164
**Knowledge frameworks**						
Total	693	387 505 702	482	0.411 (0.408-0.415)	0.152 (0.113-0.191)	
Centers for translational and computational research	144	181 240 503	670	0.418 (0.413-0.422)	0.208 (0.132-0.283)	0.085
Ontology generation	99	64 277 669	212	0.428 (0.414-0.441)	0.078 (0.028-0.129)	0.312
Knowledge bases	176	61 076 599	471	0.383 (0.374-0.392)	0.834 (−0.233-1.902)	0.064
Knowledge representation and reasoning	122	37 427 050	395	0.407 (0.393-0.420)	0.585 (0.337-0.833)	0.074
Literature review	76	25 836 844	197	0.415 (0.392-0.437)	0.104 (0.053-0.155)	0.212
Intelligent search engines and data visualization	76	17 647 037	174	0.393 (0.368-0.418)	0.098 (0.064-0.133)	0.037
**Biochemical analysis**						
Total	788	364 738 899	246	0.393 (0.388-0.398)	0.109 (0.086-0.131)	
Protein structure and binding prediction	130	94 382 477	417	0.412 (0.403-0.420)	0.054 (0.022-0.086)	0.061
Drug discovery	156	84 737 388	116	0.404 (0.390-0.419)	0.115 (0.079-0.152)	0.068
Other chemical compound characterization	159	68 832 086	191	0.377 (0.365-0.390)	0.220 (0.166-0.275)	0.078
Mass spectroscopy	145	46 167 945	269	0.402 (0.388-0.417)	0.077 (0.052-0.102)	0.162
Cell signaling pathways	117	36 077 544	241	0.368 (0.352-0.383)	0.181 (0.132-0.230)	0.151
Small molecule interactions	81	34 541 459	181	0.337 (0.320-0.354)	0.409 (0.256-0.562)	0.111
**Infectious disease/immunologic**						
Total	655	317 711 916	216	0.422 (0.415-0.429)	0.366 (0.327-0.405)	
HIV	243	115 512 623	261	0.417 (0.406-0.428)	0.266 (0.230-0.303)	0.240
Other infectious disease	242	102 079 866	202	0.421 (0.410-0.433)	0.550 (0.511-0.589)	0.088
Immunology	170	100 119 427	179	0.429 (0.417-0.441)	0.322 (0.235-0.408)	0.072
**Cancer**						
Total	799	307 395 742	209	0.424 (0.417-0.430)	0.164 (0.143-0.184)	
Other	375	165 458 242	201	0.436 (0.427-0.445)	0.187 (0.167-0.207)	0.141
Breast	297	97 856 805	215	0.406 (0.396-0.416)	0.130 (0.100-0.160)	0.244
Prostate	127	44 080 695	228	0.424 (0.410-0.439)	0.111 (0.066-0.156)	0.242
**Language and communication**						
Total	785	294 191 413	244	0.427 (0.421-0.434)	0.259 (0.230-0.289)	
Language development and reading comprehension	271	108 380 111	157	0.420 (0.408-0.433)	0.264 (0.206-0.322)	0.101
Social media and social behavior	212	84 674 749	219	0.423 (0.410-0.436)	0.310 (0.280-0.340)	0.103
Speech	210	66 336 577	346	0.409 (0.398-0.420)	0.174 (0.147-0.202)	0.239
Interpersonal communication technologies	92	34 799 976	376	0.488 (0.472-0.504)	0.261 (0.210-0.311)	0.108
**Data types**						
Total	698	270 275 389	343	0.420 (0.414-0.426)	0.207 (0.183-0.230)	
Wearable devices and mobile technology	193	81 082 336	358	0.419 (0.408-0.429)	0.354 (0.264-0.444)	0.106
Text mining	220	80 300 898	278	0.423 (0.411-0.435)	0.060 (0.040-0.080)	0.076
Motion tracking and artifact reduction	150	60 926 617	212	0.413 (0.398-0.427)	0.694 (0.569-0.819)	0.108
Big data	135	47 965 538	594	0.423 (0.413-0.433)	0.199 (0.125-0.274)	0.174
**Patient safety**						
Total	517	193 476 745	225	0.427 (0.419-0.436)	0.403 (0.323-0.482)	
Adverse drug events/drug safety	265	93 105 852	260	0.422 (0.410-0.434)	0.238 (0.206-0.271)	0.035
Surgical planning	137	64 262 285	146	0.455 (0.437-0.474)	0.843 (0.656-1.029)	0.118
Other patient safety	115	36 108 608	274	0.414 (0.399-0.430)	0.131 (0.097-0.166)	0.088
**Population health**						
Total	385	163 133 545	236	0.424 (0.414-0.434)	0.419 (0.384-0.453)	
Older adults	171	77 836 693	370	0.430 (0.418-0.443)	0.335 (0.298-0.371)	0.191
Population health screening	151	62 881 100	98	0.406 (0.383-0.429)	0.561 (0.418-0.705)	0.087
Pediatrics	63	22 415 752	161	0.421 (0.394-0.448)	0.716 (0.590-0.842)	0.158
**Model types**						
Total	473	151 710 103	307	0.423 (0.414-0.431)	0.362 (0.301-0.423)	
Deep learning	221	72 890 312	164	0.428 (0.413-0.443)	0.725 (0.630-0.820)	0.044
Natural language processing	121	48 501 377	200	0.442 (0.427-0.457)	0.121 (0.106-0.136)	0.193
Unspecified classification models	131	30 318 414	821	0.402 (0.389-0.416)	0.086 (0.055-0.117)	0.084
**Respiratory**						
Total	310	147 146 357	221	0.413 (0.403-0.422)	0.306 (0.230-0.382)	
Asthma	93	75 131 908	159	0.441 (0.425-0.456)	0.253 (0.159-0.348)	0.408
Lung cancer and COPD	217	72 014 449	285	0.397 (0.386-0.409)	0.396 (0.320-0.471)	0.180
**Electronic health record**						
Total	296	111 660 265	489	0.431 (0.424-0.439)	0.381 (0.356-0.406)	
Electronic health record	296	111 660 265	489	0.431 (0.424-0.439)	0.381 (0.356-0.406)	0.068
**Vision**						
Total	359	110 404 148	434	0.412 (0.405-0.420)	0.337 (0.272-0.402)	
Visual processing	168	50 365 533	443	0.397 (0.386-0.408)	0.098 (0.073-0.124)	0.134
Object tracking and recognition	113	34 940 758	515	0.420 (0.407-0.432)	0.102 (0.072-0.132)	0.121
Visual impairment	78	25 097 857	306	0.442 (0.423-0.460)	0.296 (0.187-0.405)	0.122
**Endocrine**						
Total	213	91 175 183	138	0.430 (0.415-0.444)	0.217 (0.139-0.296)	
Diabetes	114	52 974 185	114	0.429 (0.408-0.450)	0.313 (0.156-0.470)	0.166
Metabolic syndrome and metabolic processes	99	38 200 998	171	0.430 (0.411-0.450)	0.149 (0.107-0.190)	0.165
**Environmental health**						
Total	203	88 913 320	1038	0.423 (0.417-0.429)	0.270 (0.236-0.303)	
Environmental health	203	88 913 320	1038	0.423 (0.417-0.429)	0.270 (0.236-0.303)	0.120
**Cardiovascular**						
Total	186	85 488 684	231	0.430 (0.418-0.442)	0.291 (0.232-0.351)	
Cardiovascular disease	186	85 488 684	231	0.430 (0.418-0.442)	0.291 (0.232-0.351)	0.048
**Injuries/trauma**						
Total	159	82 210 223	144	0.414 (0.396-0.431)	0.217 (0.154-0.280)	
Trauma	159	82 210 223	144	0.414 (0.396-0.431)	0.217 (0.154-0.280)	0.075
**Renal**						
Total	123	43 663 402	365	0.436 (0.422-0.451)	0.733 (0.637-0.829)	
Kidney disease	123	43 663 402	365	0.436 (0.422-0.451)	0.733 (0.637-0.829)	0.211
**Hepatic**						
Total	123	39 489 735	256	0.459 (0.441-0.477)	0.140 (0.103-0.177)	
Liver disease	123	39 489 735	256	0.459 (0.441-0.477)	0.140 (0.103-0.177)	0.211
**Training and education**						
Total	149	35 235 945	288	0.424 (0.406-0.441)	0.099 (0.069-0.130)	
Student training and education	149	35 235 945	288	0.424 (0.406-0.441)	0.099 (0.069-0.130)	0.156

Abbreviations: APT, approximate potential to translate; COPD, chronic obstructive pulmonary disease; EEG, electroencephalogram.

**Figure 2.**
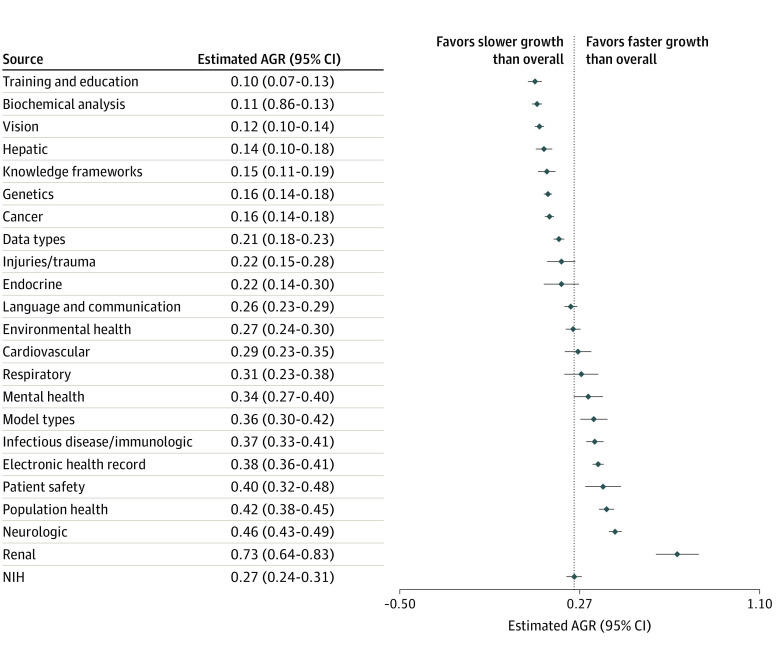
Estimated National Institutes of Health Funding Annual Growth Rate, by Category of Artificial Intelligence Applications AGR indicates annual growth rate; NIH, National Institutes of Health.

The clusters were further grouped by general application categories. Some categories were clinically focused, such as neurologic disease, cancer, and mental health, and others were technically focused, such as data types and model types. The estimated annual growth rate for NIH-funded AI research overall was 0.274 (95% CI, 0.24-0.309), with the fastest growing application categories including kidney disease (0.733; 95% CI, 0.637-0.829), neurologic disease (0.457; 95% CI, 0.429-0.485), and population health (0.419; 95% CI, 0.384-0.453) (Figure 2). Applications with low estimated annual growth rate included training and education (0.099; 95% CI, 0.069-0.130), biochemical analysis (0.109; 95% CI, 0.086-0.131), and vision (0.120; 95% CI, 0.100-0.140).

### Applications by Funding Mechanisms

The 4 most frequent funding mechanisms in the award sample were R01 (investigator-initiated research projects, 48% of all awards), U01 (research project cooperative agreements between the funding NIH institute and investigators, 4.8%), R44 (small business innovation research grants, 4.2%), and R21 (exploratory/developmental research grants, 6.8%) (eTable 7 in the Supplement). Application categories that tended to have a higher proportion of R01 grants include biochemical analysis (53.3%), genetics (55.9%), and language and communication (59.0%). The cancer (10.5%) and hepatic disease (19.5%) categories tended to have a higher proportion of U01 grants than other categories (eTable 8 and eTable 9 in the Supplement).

### Applications by Translational Impact Potential Metrics

**Figure 3.**
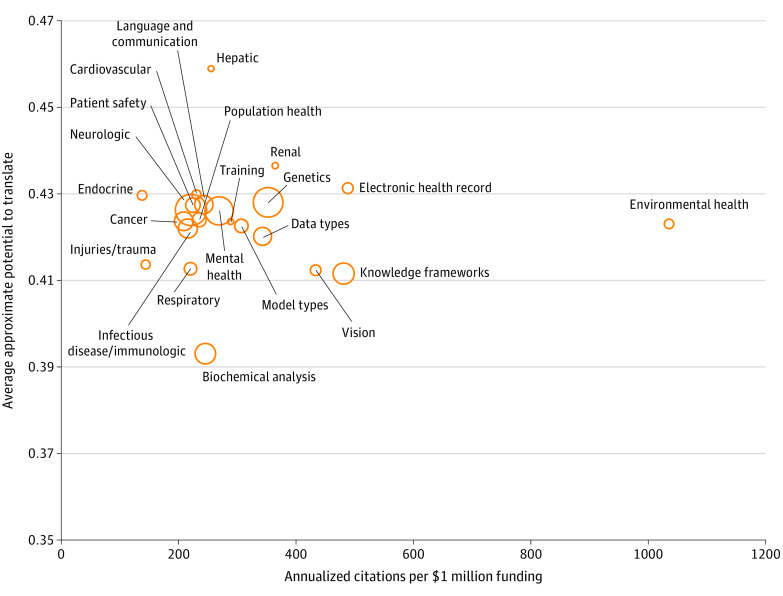
Translatability of National Institutes of Health–Funded Biomedical Research Applying Artificial Intelligence, by Category of Artificial Intelligence Applications The areas of the bubbles reflect the relative amount of National Institutes of Health research funding allocated to each category of artificial intelligence applications.

Translatability was assessed at both the specific application (Table 2) and general category (Figure 3) levels. General categories, such as liver disease (average APT, 0.459; 95% CI, 0.441-0.477), kidney disease (average APT, 0.436; 95% CI, 0.422-0.451), and the electronic health record (average APT, 0.431; 95% CI, 0.424-0.439), had high translatability. Specific applications with high APT included interpersonal communication technologies (average APT, 0.488; 95% CI, 0.472-0.504) and population genetics (average APT, 0.463; 95% CI, 0.453-0.472). The biochemical analysis category (comprising drug discovery, other chemical compound characterization, mass spectroscopy, cell signaling pathways, and small molecule interactions as applications) had the lowest translatability (average APT, 0.393; 95% CI, 0.388-0.398).

General categories with high translatability by ACOF included environmental health (ACOF, 1038), the electronic health record (ACOF, 489) and knowledge frameworks (ACOF, 482). The biochemical analysis category (ACOF, 246) was determined to be among the least translatable by ACOF as well. At both the specific application and general category levels, applications related to primary care practice were found to have the lowest translatability by ACOF. These areas of primary care included diabetes (ACOF, 114) and metabolic syndrome (ACOF, 171) in the endocrine category and population health screening in the population health category (ACOF, 98).

## Discussion

By applying unsupervised machine learning techniques to text data from NIH-funded AI research grants, we identified meaningful differences in NIH funding trends, funding types, and translational potential for various applications of AI in biomedical research. There was an 80-fold increase in annual NIH funding for AI over the 35-year study period. The terms enriched in the post-HITECH subset of awards, *ehr*, *big* (ie, big data), and *deep* (ie, deep learning), reflect how the widespread adoption of electronic medical records in the 21st century partially catalyzed this expansion by facilitating access to large, multimodal data sets for the development of AI models.^24^ Although the average APT increased between the pre- and post-HITECH periods, the ACOF decreased. This discrepancy may be explained by a decrease in the number of breakthrough articles on biomedical AI as the technologies have become more pervasive, but an increase in the potential for such technologies to create clinical value.

Identifying applications of AI with the potential to produce high returns toward the quality and efficiency of health care delivery is necessary. Some NIH institutes seem to have contributed more toward this goal than others. In our analysis, the National Center for Advancing Translational Sciences, National Institute of Dental and Craniofacial Research, National Eye Institute, and National Institute of Biomedical Imaging and Bioengineering were identified as granting highly translatable awards toward AI. The National Institute of Biomedical Imaging and Bioengineering and National Institute of Dental and Craniofacial Research have successfully implemented academic-industry partnerships to facilitate translation of AI technologies.^25^ Among the National Center for Advancing Translational Sciences core technologies are machine learning methods for prediction of chemical properties and NLP for extracting knowledge from data in rare diseases.^26^ The National Eye Institute has funded the development of an AI imaging technology to detect retinopathy of prematurity that was recently granted breakthrough status by the US Food and Drug Administration.^27^

Specific applications that were identified as having high translatable potential include environmental health and interpersonal communication technologies. Interpersonal communication technologies can aid with communication for patients with functional impairments through brain-computer interfaces, hand sign analysis, and eye tracking, among other methods.^28^ Environmental health AI can help elucidate how environmental toxin exposures contribute to the development of disease and promote preventive policy and infrastructural changes.^29,30^

Applications focused on primary care settings seemed to have lower translatability. One such application in diabetes was a proposal to generate policy recommendations that reduce preventive care disparities in patients with diabetes using Medicare claims data and Markov decision process analysis (eTable 5 in the Supplement, detecting, understanding, and reducing diabetes belt preventive care disparities). The population health screening application included studies focused on identifying cost-effective screening methods and developing personalized screening recommendations for colon cancer, among others.^31,32^ Poor translatability could be because, in the primary care setting, there is an increased need for altered patient and health care professional behavior for these types of AI technologies to make changes. Poor patient adherence to preventive health care measures and poor health care professional adoption of novel screening tools are well-recognized issues.^33,34^

Applications in the biochemical analysis category (protein structure and binding prediction, drug discovery, other chemical compound characterization, mass spectroscopy, cell signaling pathways, and small molecule interactions) were deemed less translatable by both metrics. Although such applications have an intuitively longer path to clinical utility, these are nonetheless domains in which discoveries can be made that transform our understanding of disease and result in novel methods of diagnosis and treatment. Artificial intelligence to predict protein structure and functional properties from amino acid sequence includes popular models such as AlphaFold (DeepMind), a deep neural network.^35^ Analyzing RNA sequence patterns at the cellular level with machine learning models has improved understanding of gene interactions and expression.^36,37^ Our analysis may be biased against these types of applications in favor of technologies with more immediate potential for translation.

Herein, we present, to our knowledge, a novel method for characterizing estimated biomedical research impact that uses NLP and data from NIH-awarded grants. We applied this approach to segment applications of AI in medicine and analyzed these applications with funding and citation data. The software developed for this study is open-source, making replication of these results and transfer of this method to other domains of interest straightforward. Other subjects could include an analysis of awards related to health disparities to highlight the inequities deemed most pressing based on academic interest or an analysis of what types of COVID-19–related research received the most NIH funding over the course of the pandemic.

### Limitations

This study has limitations. First, although our approach identified a number of recognized medical applications of AI, it is hindered by the lack of a standardized definition of AI. Lack of a standardized definition limits our ability to determine whether our query captured the most complete set of AI-related NIH awards or whether awards were included with aims that could be deemed unrelated to AI. Second, the k-means algorithm is an imperfect method for unsupervised clustering and there was likely varying degrees of topic overlap between the generated clusters. Conversely, the awards that were sorted into ill-defined clusters and subsequently excluded from the application analysis may have been appropriately included in a described cluster or separated into smaller defined clusters should a manual review have been performed. Third, citation counts are an imperfect proxy for research impact: citation of an academic article may also be influenced by author reputation, research domain, negative citations, and self-citations.^38,39^ Both citation-based translational impact metrics used in this study are simply proxies for the true measure of biomedical research impact: improvement in human health.

## Conclusions

Findings from this study suggest that there are numerous applications of AI in biomedical research that are receiving exponentially increasing amounts of grant funding from the NIH, demonstrating varying degrees of estimated translational impact returns. Domains of biomedical research can be categorized using NIH research grant data to understand differences in academic productivity, funding support, and clinical translation.

## Notes

Supplement. **eMethods.** Detailed Methods **eTable 1.** Framework for Defining Artificial Intelligence **eFigure 1.** Natural Language Processing Pipeline **eFigure 2.** Empiric Selection of the Number of Clusters (K) for k-Means Algorithm **eFigure 3.** NIH Funding for Artificial Intelligence by Year **eTable 2.** NIH Funding for Artificial Intelligence and Translatability by NIH Institute **eTable 3.** Validation of k-Means Generated Topics Against NIH, Condition, and Disease Categories **eTable 4.** Validation of k-Means Generated Topics Against Manual Topic Assignment **eTable 5.** Centroids for Each k-Means Generated Topic **eTable 6.** Representative Awards From Each Cluster of Artificial Intelligence Applications in Biomedical Research **eTable 7.** National Institutes of Health Funding for Artificial Intelligence by Funding Mechanism **eTable 8.** Funding Mechanism Frequencies Among National Institutes of Health–Funded Artificial Intelligence Applications in Biomedical Research **eTable 9.** Comparison of National Institutes of Health Funding Mechanism Frequency for the Four Most Common Funding Mechanisms, by Pairwise General Application Categories
