# Supplementary material for: Translatability Analysis of National Institutes of Health–Funded Biomedical Research That Applies Artificial Intelligence
Source: JAMA Netw Open. 2022 Jan 24;5(1):e2144742. doi: 10.1001/jamanetworkopen.2021.44742 (PMC8787619; doi:10.1001/jamanetworkopen.2021.44742)
Supplement: Supplement. — eMethods. Detailed Methods eTable 1. Framework for Defining Artificial Intelligence eFigure 1. Natural Language Processing Pipeline eFigure 2. Empiric Selection of the Number of Clusters (K) for k-Means Algorithm eFigure 3. NIH Funding for Artificial Intelligence by Year eTable 2. NIH Funding for Artificial Intelligence and Translatability by NIH Institute eTable 3. Validation of k-Means Generated Topics Against NIH, Condition, and Disease Categories eTable 4. Validation of k-Means Generated Topics Against Manual Topic Assignment eTable 5. Centroids for Each k-Means Generated Topic eTable 6. Representative Awards From Each Cluster of Artificial Intelligence Applications in Biomedical Research eTable 7. National Institutes of Health Funding for Artificial Intelligence by Funding Mechanism eTable 8. Funding Mechanism Frequencies Among National Institutes of Health–Funded Artificial Intelligence Applications in Biomedical Research eTable 9. Comparison of National Institutes of Health Funding Mechanism Frequency for the Four Most Common Funding Mechanisms, by Pairwise General Application Categories [file jamanetwopen-e2144742-s001.pdf]

## Supplementary Online Content

Eweje FR, Byun S, Chandra R, et al. Translatability analysis of National Institutes of Health–funded biomedical research that applies artificial intelligence. *JAMA Netw Open*. 2022;5(1):e2144742. doi:10.1001/jamanetworkopen.2021.44742

**eMethods.** Detailed Methods

**eTable 1.** Framework for Defining Artificial Intelligence

**eFigure 1.** Natural Language Processing Pipeline

**eFigure 2.** Empiric Selection of the Number of Clusters (K) for k-Means Algorithm

**eFigure 3.** NIH Funding for Artificial Intelligence by Year

**eTable 2.** NIH Funding for Artificial Intelligence and Translatability by NIH Institute

**eTable 3.** Validation of k-Means Generated Topics Against NIH,

Condition, and Disease Categories

**eTable 4.** Validation of k-Means Generated Topics Against Manual Topic Assignment

**eTable 5.** Centroids for Each k-Means Generated Topic

**eTable 6.** Representative Awards From Each Cluster of Artificial Intelligence Applications in Biomedical Research

**eTable 7.** National Institutes of Health Funding for Artificial Intelligence by Funding Mechanism

**eTable 8.** Funding Mechanism Frequencies Among National Institutes of Health–Funded Artificial Intelligence Applications in Biomedical Research

**eTable 9.** Comparison of National Institutes of Health Funding Mechanism Frequency for the Four Most Common Funding Mechanisms, by Pairwise General Application Categories

This supplementary material has been provided by the authors to give readers additional information about their work.

© 2022 Eweje FR et al. *JAMA Network Open.*

**eMethods. Detailed Methods**

(a) Defining artificial intelligence
To identify artificial intelligence-related terms for the NIH RePORTER query, key words were determined according to three major tasks that can be performed by artificial intelligence: learning and perception, knowledge representation and reasoning, and natural language processing. The search terms were methodologies and algorithms within these three tasks (**eTable 1**). When terms were known to have overlap with domains of biomedical research outside of artificial intelligence (e.g. ‘reinforcement learning’ also describes a concept in neuroscience), they were omitted with awareness that relevant awards applying these methods would likely be captured by the terms “artificial intelligence” or “machine learning”.

With this framework in mind, the following phrase was used to query the NIH RePORTER: "artificial intelligence" or "machine learning" or "deep learning" or "supervised learning" or "naive bayes" or "decision tree" or "random forest" or "support vector machine" or "K-nearest neighbor" or "K-means" or "singular value decomposition" or "apriori" or "hidden markov model" or "principal component analysis" or "hierarchical clustering" or "gaussian mixture" or "q-learning" or "markov decision process" or "artificial neural network" or "convolutional neural network" or "recurrent neural network" or "long short-term memory" or "knowledge representation" or "logical representation" or "propositional logic" or "predicate logic" or "semantic network" or "production rules" or "rule-based system" or "frame representation" or "frame language" or "frame network" or "slot-filter" or "semantic frame" or "expert system" or "natural language processing" or “named entity recognition” or “sentiment analysis” or “aspect mining” or “topic modeling” or “text mining”

(b) Cluster validation
The validity of the topics generated by the k-means algorithm was assessed by comparing to the NIH Research, Condition, and Disease Categories (RCDC) and performing rater validation of the topics. The following RCDC categories were used for this comparison: Alzheimer’s Disease, Breast Cancer, HIV/AIDS, Autism, Pain Research, Stroke, Dementia, Kidney Disease, Liver Disease, Schizophrenia, Depression, and Asthma. To assess agreement between RCDC and our k-means algorithm, we calculated the number of awards in each of the clusters of interest that received the corresponding RCDC tag (**eTable 3**). This assessment was limited only to awards granted after the RCDC tag had been implemented; for example, the “Pain Research” category was not tracked until 2012.

Manual cluster validation was performed by two authors blinded to the final award assignments. 200 awards were randomly selected from among the final 75 clusters of AI applications. Raters were instructed to assign each award to the k-means-defined topic they determined to best characterize the focus of the award’s content or methodologies. Interrater reliability was assessed using Cohen’s **κ** (**eTable 4**).

© 2022 Eweje FR et al. *JAMA Network Open.*

**eTable 1.** Framework for defining artificial intelligence

| **Task** | **Key words** |
|---|---|
| Learning and Perception | ‘machine learning’, ‘supervised learning’, ‘naive bayes’, ‘decision tree’, ‘random forest’, ‘support vector machine’, ‘k-nearest neighbor’, ‘k-means’, ‘singular value decomposition’, ‘hidden markov model’, ‘principal component analysis’, ‘hierarchical clustering’, ‘gaussian mixture’, ‘q-learning’, ‘markov decision process’, ‘artificial neural network’, ‘convolutional neural network’, ‘recurrent neural network’, ‘long short-term memory’, ‘deep learning’ |
| Knowledge Representation and Reasoning | ‘knowledge representation’, ‘logical representation’, ‘propositional logic’, ‘predicate logic’, ‘production rules’, ‘rule-based system’, ‘frame representation’, ‘frame network’, ‘slot-filter’, ‘semantic frame’, ‘expert system’, ‘bayesian network’ |
| Natural Language Processing | ‘natural language processing’, ‘named entity recognition’, ‘sentiment analysis’, ‘aspect mining’, ‘topic modeling’, ‘text mining’ |

© 2022 Eweje FR et al. *JAMA Network Open.*

**eFigure 1.** Natural language processing pipeline

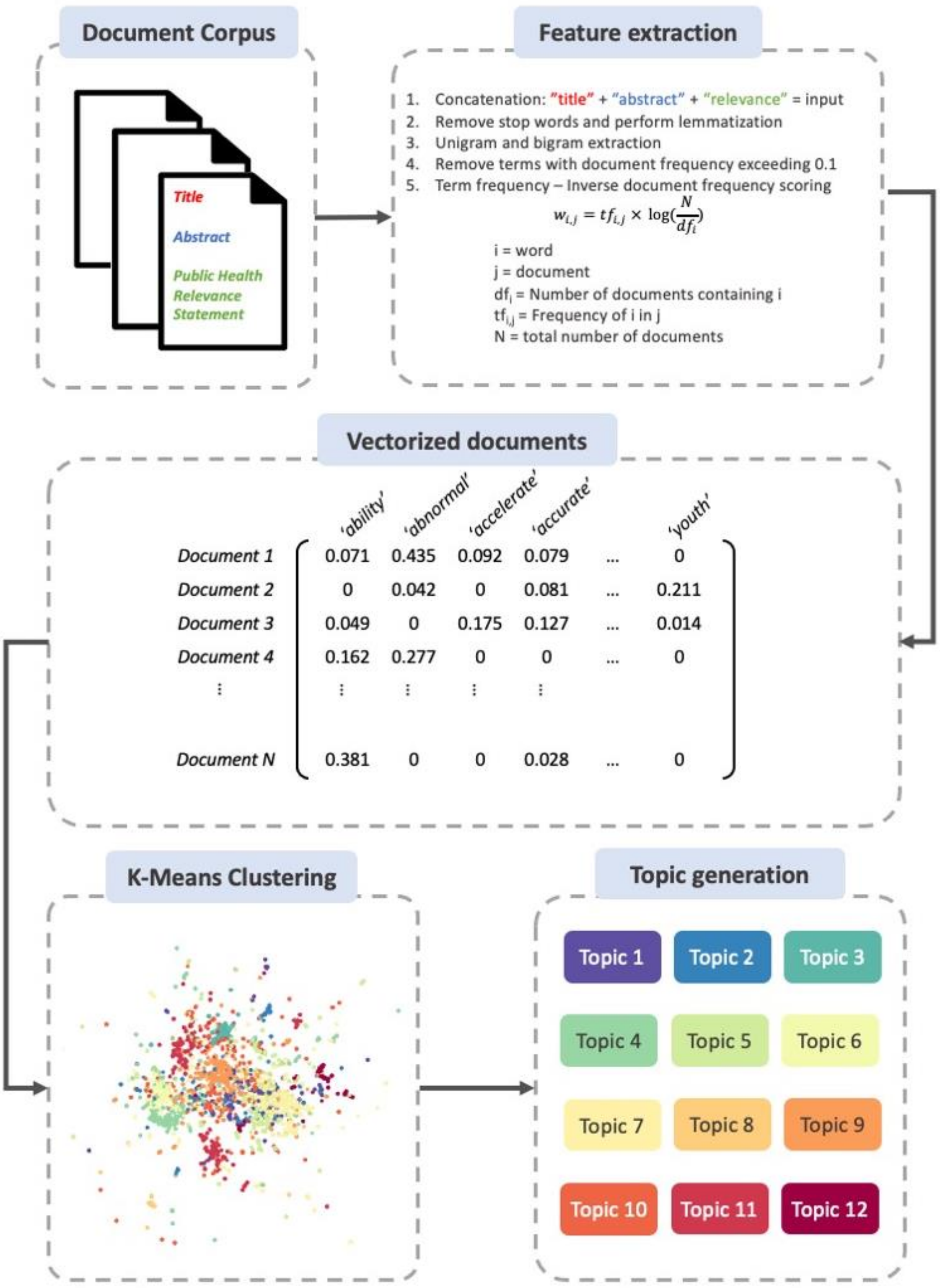


© 2022 Eweje FR et al. *JAMA Network Open.*

**eFigure 2. Empiric selection of the number of clusters (K) for k-means algorithm**. 5 trials were conducted at K of 5 through 115 at intervals of 5, inclusive. Error bars indicate 95% confidence intervals. Optimal K was selected where the additional clusters did not produce meaningfully improved silhouette scores.

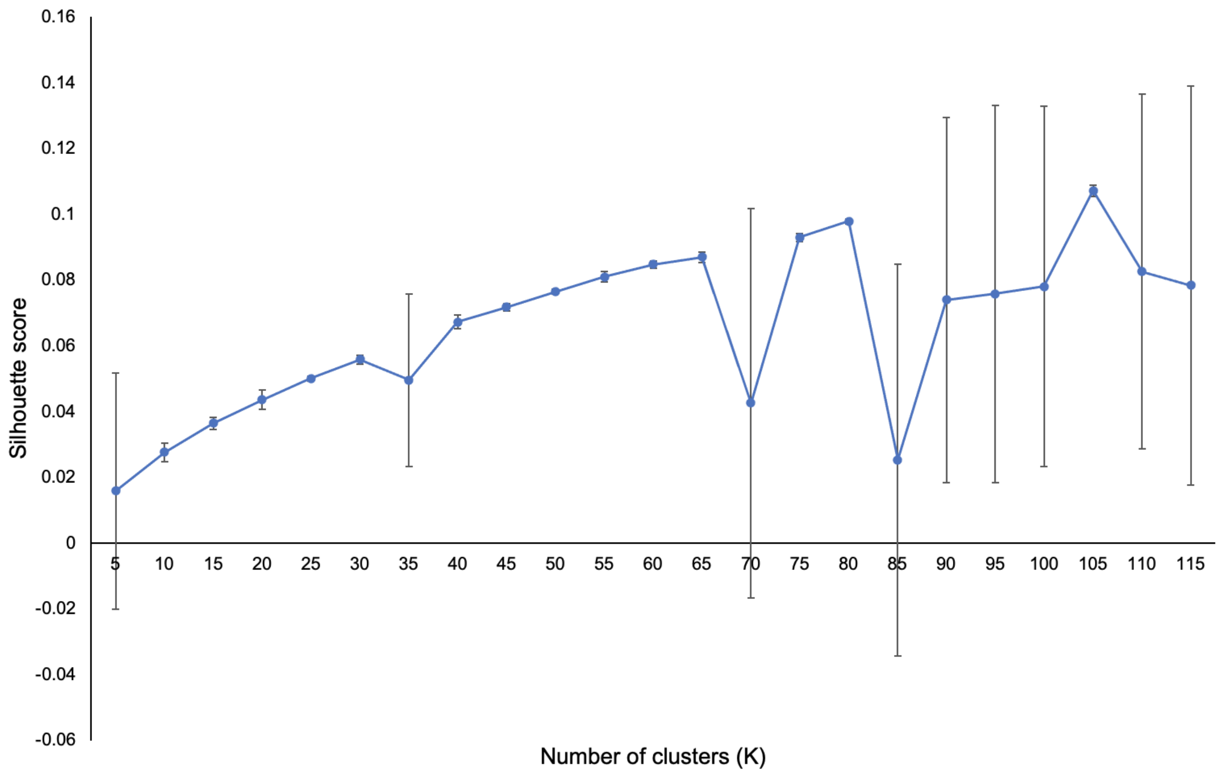


© 2022 Eweje FR et al. *JAMA Network Open.*

**eFigure 3.** NIH funding for artificial intelligence by year

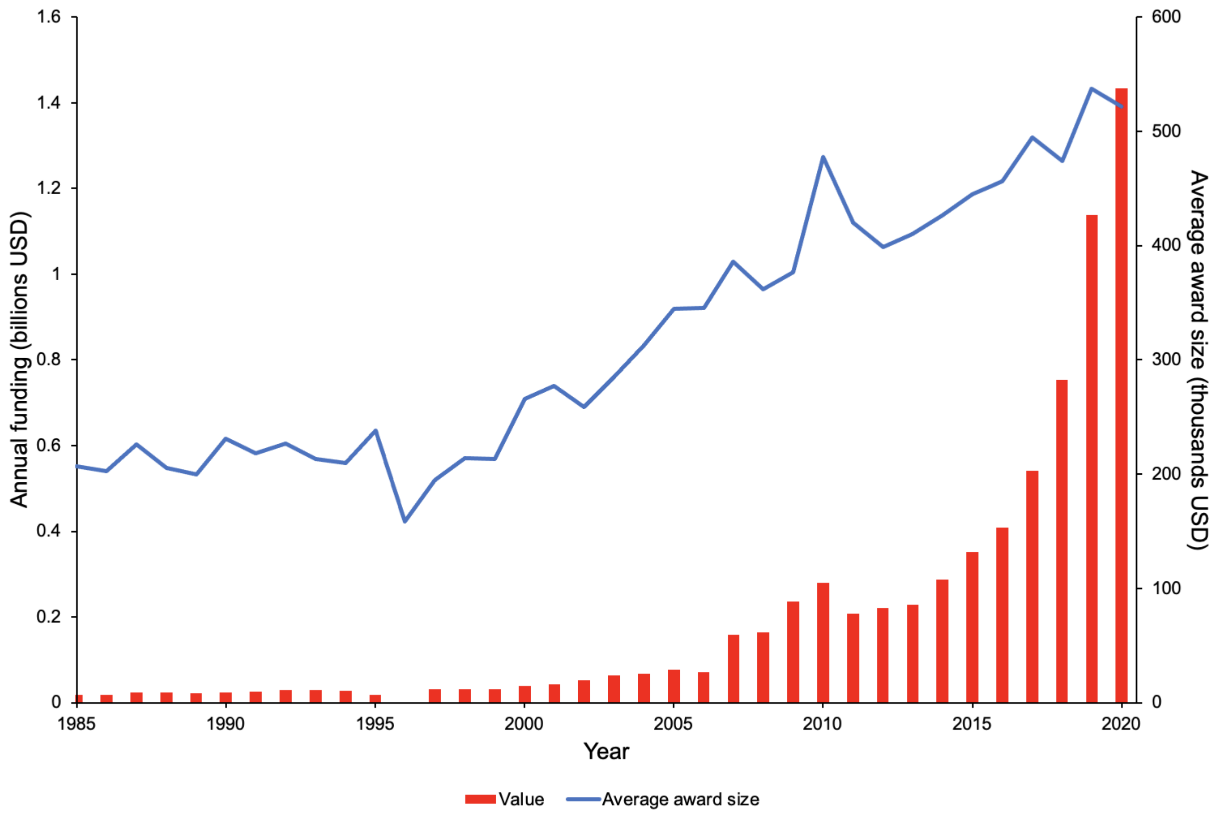


© 2022 Eweje FR et al. *JAMA Network Open.*

**eTable 2.** NIH funding for artificial intelligence and translatability by NIH institute

| Institute | Number of awards | Value of awards | Annualized citations per $1 million funding | Average Approximate Potential to Translate (95% CI) |
|---|---|---|---|---|
| National Cancer Institute (NCI) | 2124 | $919,034,802 | 88 | 0.4156 (0.4097-0.4215) |
| National Institute of General Medical Sciences (NIGMS) | 1860 | $647,946,465 | 105 | 0.4165 (0.4099-0.4231) |
| National Institute on Mental Health (NIMH) | 1745 | $768,669,654 | 49 | 0.4169 (0.4082-0.4255) |
| National Library of Medicine (NLM) | 1260 | $371,109,734 | 64 | 0.4156 (0.4051-0.4261) |
| National institute on Neurological Disorders and Stroke (NINDS) | 1080 | $474,259,337 | 56 | 0.4126 (0.4022-0.4230) |
| National Heart, Lung, and Blood Institute (NHLBI) | 1080 | $525,427,401 | 51 | 0.4079 (0.3981-0.4176) |
| National Institute on Aging (NIA) | 856 | $505,819,658 | 111 | 0.4282 (0.4208-0.4356) |
| National Human Genome Research Institute (NHGRI) | 767 | $506,754,443 | 51 | 0.4303 (0.4195-0.4412) |
| National Institute of Biomedical Imaging and Bioengineering (NIBIB) | 657 | $246,024,757 | 157 | 0.4245 (0.4157-0.4332) |
| National Institute of Child Health and Human Development (NICHD) | 647 | $225,006,972 | 131 | 0.4339 (0.4238-0.4441) |
| National Institute of Allergy and Infectious¬† Diseases (NIAID) | 602 | $304,232,560 | 55 | 0.4123 (0.3986-0.4260) |
| National Institute on Drug Abuse (NIDA) | 549 | $223,527,560 | 58 | 0.4155 (0.3995-0.4316) |
| National Institute of Diabetes and Digestive and Kidney Diseases (NIDDK) | 510 | $219,476,634 | 78 | 0.4239 (0.4106-0.4371) |
| National Institute on Deafness and Other Communication Disorders (NIDCD) | 457 | $163,826,719 | 59 | 0.4192 (0.4025-0.4359) |
| National Eye Institute (NEI) | 392 | $138,412,419 | 100 | 0.4408 (0.4263-0.4553) |
| National Institute of Environmental Health Sciences (NIEHS) | 368 | $143,433,072 | 190 | 0.4263 (0.4158-0.4368) |
| National Institute on Alcohol Abuse and Alcoholism (NIAAA) | 309 | $115,643,051 | 102 | 0.4083 (0.3911-0.4255) |
| National Institute of Arthritis and Musculoskeletal and Skin Diseases (NIAMS) | 196 | $155,387,065 | 39 | 0.4070 (0.3847-0.4294) |

© 2022 Eweje FR et al. *JAMA Network Open.*

| | | | | |
|---|---|---|---|---|
| Agency for Healthcare Research and Quality (AHRQ) | 192 | $74,020,560 | 35 | 0.3976 (0.3649-0.4303) |
| National Institute of Dental and Craniofacial Research (NIDCR) | 186 | $59,298,177 | 65 | 0.4447 (0.4167-0.4727) |
| National Center for Research Resources (NCRR) | 173 | $98,270,039 | 120 | 0.3927 (0.3780-0.4075) |
| National Institute of Nursing Research (NINR) | 152 | $51,095,747 | 68 | 0.4335 (0.4028-0.4642) |
| National Center for Advancing Translational Sciences (NCATS) | 109 | $76,007,518 | 392 | 0.4106 (0.4007-0.4206) |
| Office of the Director, NIH (OD) | 103 | $70,309,304 | 28 | 0.3804 (0.3483-0.4125) |
| National Center for Complementary and Integrative Health (NCCIH) | 73 | $37,544,567 | 67 | 0.4471 (0.4045-0.4896) |
| National Institute on Minority Health and Health Disparities (NIMHD) | 51 | $20,592,544 | 16 | 0.3674 (0.2858-0.4491) |
| National Institute of Occupational Safety and Health (NIOSH) | 35 | $4,307,437 | 3 | 0.2500 (0.0771-0.4229) |
| Food and Drug Administration (FDA) | 27 | $7,455,759 | 4 | 0.1885 (0.0585-0.3184) |
| Fogarty International Center (FIC) | 22 | $4,667,473 | 60 | 0.3758 (0.2982-0.4534) |
| National Center for Emerging and Zoonotic Infectious Diseases (NCEZID) | 11 | $5,688,113 | 0 | - |
| National Center for Chronic Disease Prevention and Health Promotion (NCCDPHP) | 9 | $2,607,109 | 9 | 0.5167 (0.0593-0.9740) |
| National Center for Injury Prevention and Control (NCIPC) | 6 | $1,294,355 | 2 | 0.75 |
| National Center on Birth Defects and Developmental Disabilities (NCBDDD) | 5 | $2,194,730 | 10 | 0.3250 (-0.0936-0.7436) |
| National Institute of Diabetes and Digestive and Kidney Diseases (NIDDK) | 4 | $362,466 | 177 | 0.1383 (0.0959-0.1808) |
| Public Health practice Program Office (PHPPO) | 4 | $2,020,406 | 43 | 0.4526 (0.2999-0.6054) |
| Central Institute for the Deaf (CID) | 2 | $782,492 | 93 | 0.4286 (0.2580-0.5992) |
| Agency for Toxic Substances and DIsease Registry (ATSDR) | 2 | $1,000,000 | 17 | 0.5 |
| Office of the Director, Centers for Disease Control and Prevention (ODCDC) | 2 | $3,159,933 | 3 | 0.5000 (-0.3820-1.3820) |

© 2022 Eweje FR et al. *JAMA Network Open.*

| | | | | |
|---|---|---|---|---|
| National Center for Health Statistics (NCHS) | 1 | $37,800 | 0 | - |
| National Center for Immunization and Respiratory Diseases (NCIRD) | 1 | $371,721 | 0 | 0.05 |

© 2022 Eweje FR et al. *JAMA Network Open.*

**eTable 3.** Validation of k-means generated topics against NIH, Condition, and Disease Categories (RCDC)

| k-means Cluster | RCDC category | Year RCDC category was added | Post-RCDC category addition awards in paired cluster | k-means-RCDC matched awards (% Matched) |
|---|---|---|---|---|
| Alzheimer's Disease | Alzheimer's Disease | 2008 | 321 | 293 (91.3%) |
| Breast Cancer | Breast Cancer | 2008 | 297 | 196 (66.0%) |
| HIV | HIV/AIDS | 2008 | 243 | 183 (75.3%) |
| Autism | Autism | 2008 | 192 | 177 (92.2%) |
| Pain | Pain Research | 2012 | 188 | 148 (78.7%) |
| Stroke | Stroke | 2008 | 176 | 138 (78.4%) |
| Dementia | Dementia | 2008 | 148 | 127 (85.8%) |
| Kidney Disease | Kidney Disease | 2008 | 123 | 109 (88.6%) |
| Liver Disease | Liver Disease | 2008 | 123 | 92 (74.8%) |
| Schizophrenia | Schizophrenia | 2008 | 113 | 75 (66.4%) |
| Depression | Depression | 2015 | 104 | 64 (61.5%) |
| Asthma | Asthma | 2008 | 93 | 74 (79.6%) |
| Suicide | Suicide | 2008 | 79 | 75 (94.9%) |

© 2022 Eweje FR et al. *JAMA Network Open.*

**eTable 4.** Validation of k-means generated topics against manual topic assignment

| **Comparison** | **Cohen's κ** | **Agreement** |
|---|---|---|
| Rater 1 vs. Algorithm | 0.375 | 0.385 |
| Rater 2 vs. Algorithm | 0.487 | 0.495 |
| Rater 1 vs. Rater 2 | 0.612 | 0.62 |

© 2022 Eweje FR et al. *JAMA Network Open.*

**eTable 5.** Centroids for each k-means generated topic

| **Application** | **Centroids** |
|---|---|
| **Neurologic** | |
| Alzheimer's disease | ['ad', 'alzheimer', 'alzheimer disease', 'dementia', 'aging', 'progression', 'pathology', 'longitudinal', 'neuroimaging', 'clinical trial', 'stage', 'mri', 'biomarker', 'impairment', 'participant'] |
| Neural circuits | ['neuron', 'circuit', 'neuronal', 'cortex', 'cortical', 'stimulus', 'cell type', 'animal', 'connectivity', 'motor', 'recording', 'property', 'neuroscience', 'mouse', 'cellular'] |
| Other dementia | ['dementia', 'alzheimer', 'alzheimer disease', 'pathology', 'ad', 'person', 'aging', 'mri', 'older', 'impairment', 'participant', 'risk factor', 'symptom', 'stage', 'people'] |
| Stroke | ['stroke', 'recovery', 'acute', 'blood', 'disability', 'motor', 'impairment', 'effectiveness', 'movement', 'deficit', 'mri', 'agent', 'lesion', 'flow', 'difference'] |
| Motor function | ['motor', 'stimulation', 'movement', 'recording', 'cortical', 'circuit', 'device', 'parameter', 'injury', 'cortex', 'array', 'action', 'sensor', 'deep', 'activation'] |
| Memory | ['memory', 'working', 'circuit', 'deficit', 'representation', 'cortex', 'stimulation', 'neuronal', 'fmri', 'animal', 'eeg', 'event', 'alzheimer', 'cortical', 'schizophrenia'] |
| EEG | ['eeg', 'recording', 'neuroscience', 'sleep', 'event', 'monitoring', 'reliable', 'resolution', 'fmri', 'cortical', 'spatial', 'period', 'mri', 'collected', 'hospital'] |
| Sleep | ['sleep', 'eeg', 'cortical', 'cardiovascular', 'profile', 'recording', 'marker', 'event', 'memory', 'device', 'physiological', 'mouse', 'movement', 'regulation', 'parameter'] |
| **Genetics** | |
| Regulatory genetics | ['regulatory', 'regulation', 'gene expression', 'element', 'cellular', 'dna', 'cell type', 'variant', 'binding', 'variation', 'normal', 'datasets', 'organization', 'assay', 'sequencing'] |
| Clinically significant genetic variation | ['variant', 'variation', 'sequencing', 'trait', 'genetics', 'causal', 'mutation', 'dna', 'genomics', 'regulatory', 'statistical method', 'interpretation', 'gene expression', 'mapping', 'influence'] |
| Molecular genetics | ['dna', 'sequencing', 'gene expression', 'binding', 'specie', 'tumor', 'site', 'profile', 'global', 'progression', 'variation', 'stage', 'base', 'regulatory', 'derived'] |
| Population genetics | ['snp', 'susceptibility', 'variant', 'gene expression', 'dna', 'candidate', 'american', 'environmental', 'inference', 'wide', 'variation', 'selection', 'age', 'cardiovascular', 'characterization'] |
| Familial genetics | ['family', 'genomics', 'implementation', 'child', 'communication', 'susceptibility', 'environmental', 'syndrome', 'history', 'implement', 'prevention', 'domain', 'dna', 'service', 'long'] |
| Gene mapping | ['mapping', 'trait', 'genetics', 'map', 'variant', 'marker', 'variation', 'powerful', 'sequencing', 'score', 'power', 'size', 'statistical method', 'physical', 'involved'] |
| Mouse modeling | ['mouse', 'gene expression', 'animal', 'shape', 'candidate', 'normal', 'dna', 'profile', 'resolution', 'vivo', 'neuron', 'susceptibility', 'assay', 'motion', 'chronic'] |
| Functional mutations | ['mutation', 'variant', 'regulatory', 'dna', 'cellular', 'consequence', 'binding', 'sequencing', 'gene expression', 'regulation', 'computational method', 'variation', 'candidate', 'genomics', 'aid'] |
| RNA analysis | ['rna', 'sequencing', 'element', 'long', 'regulatory', 'regulation', 'cellular', 'binding', 'mapping', 'generation', 'dna', 'molecule', 'recognition', 'comparison', 'example'] |

© 2022 Eweje FR et al. *JAMA Network Open.*

| | |
|---|---|
| **Mental Health** | |
| Pain | ['pain', 'chronic', 'acute', 'syndrome', 'low', 'woman', 'symptom', 'influence', 'collaboration', 'surgery', 'sensitivity', 'substance', 'hypothesize', 'award', 'physiological'] |
| Autism spectrum disorder | ['asd', 'autism', 'child', 'connectivity', 'spectrum', 'social', 'developmental', 'infant', 'impairment', 'deficit', 'heterogeneity', 'core', 'mri', 'communication', 'longitudinal'] |
| Alcohol use | ['alcohol', 'exposure', 'child', 'spectrum', 'consortium', 'deficit', 'adolescent', 'youth', 'core', 'feedback', 'period', 'month', 'substance', 'prevention', 'site'] |
| Other mental health | ['mental', 'mental health', 'illness', 'psychiatric', 'depression', 'neuroscience', 'symptom', 'social', 'variation', 'environmental', 'schizophrenia', 'neuroimaging', 'variable', 'influence', 'child'] |
| Adolescent psychiatry | ['adolescent', 'substance', 'youth', 'mobile', 'symptom', 'child', 'alcohol', 'prevention', 'difference', 'consortium', 'developmental', 'career', 'family', 'social', 'skill'] |
| Other child development | ['infant', 'child', 'developmental', 'speech', 'acquisition', 'growth', 'learn', 'age', 'input', 'month', 'deficit', 'social', 'stress', 'mri', 'exposure'] |
| Depression | ['depression', 'symptom', 'circuit', 'personalized', 'mobile', 'fmri', 'sensor', 'negative', 'positive', 'construct', 'mental', 'participant', 'adolescent', 'collected', 'mental health'] |
| Suicidality | ['suicide', 'death', 'risk factor', 'prevention', 'youth', 'adolescent', 'predictor', 'ehr', 'psychiatric', 'mental', 'health care', 'prior', 'setting', 'healthcare', 'social'] |
| Schizophrenia | ['schizophrenia', 'deficit', 'illness', 'abnormality', 'symptom', 'fmri', 'temporal', 'psychiatric', 'paradigm', 'mental', 'dysfunction', 'neuroimaging', 'candidate', 'cortex', 'connectivity'] |
| **Knowledge frameworks** | |
| Centers for translational and computational research | ['core', 'informatics', 'infrastructure', 'continue', 'consortium', 'neuroscience', 'service', 'collaborative', 'bioinformatics', 'access', 'domain', 'alcohol', 'capability', 'genetics', 'scientist'] |
| Ontology generation | ['ontology', 'annotation', 'integration', 'representation', 'infrastructure', 'scientist', 'collaborative', 'national', 'term', 'informatics', 'consortium', 'context', 'data set', 'essential', 'domain'] |
| Knowledge bases | ['base', 'representation', 'artificial intelligence', 'intelligence', 'artificial', 'interface', 'domain', 'aid', 'capability', 'continue', 'collaborative', 'national', 'interpretation', 'access', 'variation'] |
| Knowledge representation and reasoning | ['representation', 'concept', 'cortex', 'world', 'theory', 'action', 'issue', 'basic', 'temporal', 'memory', 'planning', 'visual', 'fmri', 'neuroscience', 'domain'] |
| Literature review | ['review', 'systematic', 'literature', 'annotation', 'text', 'screening', 'alternative', 'automatically', 'validation', 'best', 'wa', 'necessary', 'clinical trial', 'report', 'able'] |
| Intelligent search engines and data visualization | ['scientist', 'learn', 'building', 'genomics', 'generated', 'bioinformatics', 'size', 'data set', 'engineering', 'career', 'step', 'rapidly', 'aspect', 'heterogeneity', 'inference'] |
| **Biochemical analysis** | |
| Protein structure and binding prediction | ['site', 'annotation', 'binding', 'specie', 'regulatory', 'element', 'location', 'capability', 'core', 'regulation', 'dna', 'access', 'basic', 'spectrum', 'global'] |

© 2022 Eweje FR et al. *JAMA Network Open.*

| | |
|---|---|
| Drug discovery | ['compound', 'agent', 'product', 'molecule', 'chemical', 'assay', 'screening', 'active', 'vivo', 'spectrum', 'small', 'infection', 'candidate', 'required', 'increasing'] |
| Other chemical compound characterization | ['chemical', 'assay', 'environmental', 'molecule', 'capability', 'animal', 'compound', 'substance', 'variety', 'signaling', 'small', 'cellular', 'mass', 'property', 'specie'] |
| Mass spectroscopy | ['mass', 'spectrum', 'characterization', 'sensitivity', 'chemical', 'classification', 'assay', 'molecule', 'lesion', 'breast', 'compare', 'marker', 'screening', 'capability', 'aid'] |
| Cell signaling pathways | ['binding', 'site', 'receptor', 'molecule', 'specificity', 'dna', 'regulatory', 'simulation', 'chemical', 'signaling', 'recognition', 'datasets', 'domain', 'interface', 'improvement'] |
| Small molecule interactions | ['molecule', 'small', 'chemical', 'genetics', 'binding', 'immune', 'receptor', 'device', 'cellular', 'property', 'resolution', 'screening', 'candidate', 'produce', 'systematic'] |
| **Infectious disease/Immunologic** | |
| HIV | ['hiv', 'infection', 'prevention', 'aid', 'resistance', 'woman', 'participant', 'social', 'substance', 'impairment', 'person', 'site', 'dna', 'people', 'efficacy'] |
| Other infectious disease | ['infection', 'host', 'resistance', 'immune', 'hospital', 'assay', 'blood', 'candidate', 'rapid', 'people', 'death', 'specie', 'global', 'severe', 'healthcare'] |
| Immunology | ['immune', 'host', 'infection', 'receptor', 'blood', 'signaling', 'adaptive', 'adverse', 'regulatory', 'signature', 'monitoring', 'assay', 'resistance', 'profile', 'event'] |
| **Cancer** | |
| Other cancer | ['tumor', 'progression', 'survival', 'normal', 'targeted', 'immune', 'vivo', 'mri', 'mutation', 'resistance', 'marker', 'heterogeneity', 'parameter', 'data analysis', 'signature'] |
| Breast cancer | ['breast', 'breast cancer', 'woman', 'tumor', 'biopsy', 'screening', 'lesion', 'receptor', 'decision support', 'early detection', 'stage', 'sensitivity', 'mass', 'positive', 'biomarker'] |
| Prostate cancer | ['prostate', 'biopsy', 'tumor', 'mri', 'marker', 'progression', 'clinically', 'radiation', 'vivo', 'planning', 'breast', 'modality', 'screening', 'negative', 'active'] |
| **Language and communication** | |
| Language development and reading comprehension | ['child', 'age', 'developmental', 'impairment', 'acquisition', 'autism', 'young', 'pediatric', 'symptom', 'deficit', 'artificial', 'social', 'family', 'skill', 'mobile'] |
| Social media and social behavior | ['social', 'access', 'young', 'influence', 'substance', 'mobile', 'content', 'status', 'prevention', 'animal', 'emerging', 'national', 'mining', 'hospital', 'skill'] |
| Speech | ['speech', 'motor', 'communication', 'impairment', 'representation', 'recognition', 'movement', 'child', 'device', 'normal', 'spectrum', 'error', 'progress', 'shape', 'person'] |
| Interpersonal communication technologies | ['communication', 'interface', 'speech', 'impairment', 'device', 'motor', 'cortex', 'people', 'clinician', 'provider', 'access', 'severe', 'participant', 'increasing', 'clinical trial'] |
| **Data types** | |

© 2022 Eweje FR et al. *JAMA Network Open.*

| | |
|---|---|
| Wearable devices and mobile technology | ['smoking', 'physical activity', 'physical', 'physician', 'month', 'participant', 'mobile', 'real', 'end', 'sensor', 'american', 'low', 'report', 'effectiveness', 'randomized'] |
| Text mining | ['text', 'literature', 'mining', 'natural language', 'language processing', 'access', 'search', 'report', 'content', 'annotation', 'ontology', 'extract', 'domain', 'concept', 'scientist'] |
| Motion tracking and artifact reduction | ['cardiac', 'heart', 'motion', 'event', 'score', 'failure', 'death', 'procedure', 'mri', 'cardiovascular', 'recording', 'flow', 'normal', 'radiation', 'shape'] |
| Big data | ['big', 'big data', 'datasets', 'scientist', 'bioinformatics', 'genomics', 'data analysis', 'student', 'integration', 'initiative', 'fmri', 'precision', 'neuroscience', 'world', 'biomarker'] |
| **Patient safety** | |
| Adverse drug events/drug safety | ['safety', 'surveillance', 'event', 'adverse', 'medication', 'electronic', 'mortality', 'natural language', 'language processing', 'hospital', 'report', 'monitoring', 'healthcare', 'health care', 'temporal'] |
| Surgical planning | ['surgical', 'surgery', 'procedure', 'skill', 'planning', 'accurately', 'shape', 'monitoring', 'ct', 'effectiveness', 'significantly', 'candidate', 'tumor', 'safety', 'vivo'] |
| Other patient safety | ['error', 'safety', 'digital', 'electronic', 'event', 'simulation', 'estimate', 'reducing', 'monitoring', 'healthcare', 'report', 'setting', 'clinician', 'concept', 'detect'] |
| **Population health** | |
| Older adults | ['older', 'older adult', 'aging', 'physical', 'person', 'chronic', 'health care', 'age', 'physical activity', 'social', 'provider', 'depression', 'cardiovascular', 'monitoring', 'clinician'] |
| Population health screening | ['screening', 'woman', 'early detection', 'mortality', 'ct', 'lesion', 'positive', 'electronic', 'interpretation', 'united state', 'united', 'detect', 'candidate', 'implementation', 'guideline'] |
| Pediatrics | ['pediatric', 'child', 'digital', 'hospital', 'decision support', 'ehr', 'acute', 'mri', 'implementation', 'health care', 'electronic', 'chronic', 'clinical trial', 'national', 'conduct'] |
| **Model types** | |
| Deep learning | ['lesion', 'deep', 'deep learning', 'mri', 'progression', 'biopsy', 'interpretation', 'digital', 'neural network', 'classification', 'pathology', 'learning algorithm', 'learn', 'longitudinal', 'learning method'] |
| Natural language processing | ['nlp', 'text', 'natural language', 'language processing', 'electronic', 'extract', 'report', 'domain', 'setting', 'translational', 'informatics', 'health record', 'decision support', 'electronic health', 'automatically'] |
| Unspecified classification models | ['classification', 'neural network', 'criterion', 'family', 'implement', 'variable', 'domain', 'procedure', 'data set', 'predicting', 'datasets', 'selection', 'statistical method', 'annotation', 'traditional'] |
| **Respiratory** | |
| Asthma | ['asthma', 'child', 'exposure', 'lung', 'chronic', 'candidate', 'immune', 'gene expression', 'pediatric', 'severe', 'environmental', 'variant', 'healthcare', 'neural network', 'school'] |
| Lung cancer and COPD | ['lung', 'ct', 'screening', 'death', 'survival', 'chronic', 'smoking', 'positive', 'injury', 'snp', 'susceptibility', 'biomarker', 'progression', 'marker', 'test hypothesis'] |
| **Electronic health record** | |

© 2022 Eweje FR et al. *JAMA Network Open.*

| | |
|---|---|
| Electronic health record | ['ehr', 'electronic', 'health record', 'electronic health', 'informatics', 'healthcare', 'natural language', 'clinical data', 'clinician', 'decision support', 'provider', 'language processing', 'physician', 'nlp', 'hospital'] |
| **Vision** | |
| Visual processing | ['visual', 'stimulus', 'cortex', 'shape', 'vision', 'cortical', 'object', 'property', 'space', 'attention', 'input', 'neuron', 'circuit', 'representation', 'stimulation'] |
| Object tracking and recognition | ['object', 'visual', 'recognition', 'action', 'theory', 'concept', 'movement', 'representation', 'memory', 'input', 'attention', 'age', 'loss', 'interface', 'cortex'] |
| Visual impairment | ['vision', 'visual', 'loss', 'low', 'device', 'people', 'property', 'early detection', 'motion', 'normal', 'sensor', 'engineering', 'person', 'laboratory', 'student'] |
| **Endocrine** | |
| Diabetes | ['diabetes', 'chronic', 'physical activity', 'clinical trial', 'achieve', 'american', 'decision support', 'consortium', 'prevention', 'body', 'people', 'blood', 'physical', 'aid', 'risk factor'] |
| Metabolic syndrome and metabolic processes | ['metabolic', 'syndrome', 'mass', 'host', 'physical', 'cardiovascular', 'spectrum', 'compound', 'targeted', 'trait', 'signature', 'age', 'resolution', 'physical activity', 'collaboration'] |
| **Environmental health** | |
| Environmental health | ['exposure', 'environmental', 'chemical', 'risk factor', 'career', 'child', 'period', 'mouse', 'animal', 'prevention', 'dna', 'estimate', 'cardiovascular', 'developmental', 'social'] |
| **Cardiovascular** | |
| Cardiovascular disease | ['heart', 'stress', 'failure', 'cardiovascular', 'cardiac', 'physician', 'parameter', 'death', 'mortality', 'symptom', 'blood', 'candidate', 'clinically', 'hospital', 'ct'] |
| **Injuries/trauma** | |
| Trauma | ['injury', 'recovery', 'acute', 'neurological', 'motor', 'translational', 'death', 'disability', 'loss', 'blood', 'mri', 'syndrome', 'people', 'correlate', 'physiological'] |
| **Renal** | |
| Kidney disease | ['kidney', 'chronic', 'progression', 'injury', 'survival', 'biopsy', 'acute', 'syndrome', 'effectiveness', 'failure', 'mortality', 'cardiovascular', 'heterogeneity', 'national', 'award'] |
| **Hepatic** | |
| Liver disease | ['liver', 'injury', 'stage', 'mouse', 'biomarker', 'radiation', 'activation', 'motion', 'tumor', 'alcohol', 'survival', 'chronic', 'early detection', 'signature', 'biopsy'] |
| **Training and education** | |
| Student training and education | ['student', 'school', 'course', 'neuroscience', 'skill', 'scientist', 'laboratory', 'career', 'engineering', 'national', 'institute', 'grant', 'content', 'basic', 'artificial'] |

© 2022 Eweje FR et al. *JAMA Network Open.*

**eTable 6.** Representative awards from each cluster of artificial intelligence applications in biomedical research. The 5 awards with maximum sample silhouette score are displayed to represent each cluster. When an award had been renewed over multiple years, the most recent award was listed.

| Description | Title | Activity | Organization | Year |
|---|---|---|---|---|
| Adolescent psychiatry | ABCD-USA Consortium:Research Project | U01 | UNIVERSITY OF PITTSBURGH AT PITTSBURGH | 2018 |
| Adolescent psychiatry | ABCD-USA Consortium: Research Project | U01 | UNIVERSITY OF CALIFORNIA, SAN DIEGO | 2019 |
| Adolescent psychiatry | Using Machine Learning Approaches to Examine Emotion-Related Brain Activity and Substance Use Among Adolescents | F31 | GEORGE MASON UNIVERSITY | 2020 |
| Adolescent psychiatry | The Gist of Hot and Cold Cognition in Adolescents Risky Decision Making | R01 | CORNELL UNIVERSITY | 2013 |
| Adolescent psychiatry | ABCD-USA Consortium: Data Analysis Center | U24 | UNIVERSITY OF CALIFORNIA, SAN DIEGO | 2019 |
| Adverse drug events/drug safety | Mapping the Drugome: predictive network approaches to drug safety surveillance | R00 | BOSTON CHILDREN'S HOSPITAL | 2014 |
| Adverse drug events/drug safety | Coupling Results Data¬†from¬†ClinicalTrials.gov¬†and¬†Bibliographic Databases¬†to Accelerate Evidence Synthesis | R01 | BOSTON CHILDREN'S HOSPITAL | 2020 |
| Adverse drug events/drug safety | Mining health data for drug safety profiles | R01 | STANFORD UNIVERSITY | 2017 |
| Adverse drug events/drug safety | Distributed, Collaborative Intelligent Agents for Proactive Post-Marketing Drug S | R21 | WAYNE STATE UNIVERSITY | 2009 |
| Adverse drug events/drug safety | Preclinical predictive markers of post-approval drug safety | R01 | BOSTON CHILDREN'S HOSPITAL | 2011 |
| Alcohol use | How Cannabis Use Affects Alcohol Treatment Outcomes | R21 | PUBLIC HEALTH INSTITUTE | 2016 |
| Alcohol use | : Complex systems analysis of the impact of alcohol on bone in non-human primates | R01 | OREGON STATE UNIVERSITY | 2020 |
| Alcohol use | Alcohol Misuse: An Independent Risk Factor that Increases the Incidence and Severity of COVID-19 | R24 | RUSH UNIVERSITY MEDICAL CENTER | 2020 |
| Alcohol use | Ambulatory assessment of alcohol use, mood dysregulation, and alcohol craving | R21 | UNIVERSITY OF MISSOURI-COLUMBIA | 2014 |
| Alcohol use | Estimating BrAC/BAC from Transdermal Alcohol: Combining First-Principles Physiological Models with Machine-Learning to Create Software to Optimally Process and Quantitatively Interpret Biosensor Data | R01 | UNIVERSITY OF SOUTHERN CALIFORNIA | 2020 |
| Alzheimer's disease | Computational Prediction and Functional Validation of Novel Risk Loci of Alzheimer's Disease | R56 | EMORY UNIVERSITY | 2018 |
| Alzheimer's disease | Circadian Organization and Disorder in Alzheimer's Disease | R01 | UNIVERSITY OF PENNSYLVANIA | 2020 |
| Alzheimer's disease | Using connectomics to characterize risk for Alzheimer's Disease | R01 | MEDICAL UNIVERSITY OF SOUTH CAROLINA | 2020 |
| Alzheimer's disease | Drug repurposing for Alzheimer's disease using structural systems pharmacology. | R01 | HUNTER COLLEGE | 2020 |
| Alzheimer's disease | DISCOURSE AND EVERYDAY REMEMBERING | R01 | UNIVERSITY OF PITTSBURGH AT PITTSBURGH | 1990 |

© 2022 Eweje FR et al. *JAMA Network Open.*

| | | | | |
|---|---|---|---|---|
| Asthma | Nasal biomarkers of asthma | R01 | ICAHN SCHOOL OF MEDICINE AT MOUNT SINAI | 2019 |
| Asthma | Multi-Omics Analysis of the Association of Polyunsaturated Fatty Acids with Asthma and Allergy in Childhood | K08 | BRIGHAM AND WOMEN'S HOSPITAL | 2020 |
| Asthma | Asthma ascertainment and characterization through electronic health records | R01 | MAYO CLINIC ROCHESTER | 2017 |
| Asthma | AUTOMATED SYSTEM FOR TELEPHONE HOME MANAGEMENT OF ASTHMA | R43 | BETTER CONTROL MEDICAL COMPUTERS (BCMC) | 1997 |
| Asthma | Clustering to identify novel phenotypes in childhood asthma | F30 | UNIVERSITY OF WISCONSIN-MADISON | 2013 |
| Autism spectrum disorder | Neurobehavioral Investigation of Tactile Features in Autism Spectrum Disorders | K01 | VANDERBILT UNIVERSITY | 2014 |
| Autism spectrum disorder | Genetic Architecture of Autisms without Intellectual Disability | R21 | UNIVERSITY OF PENNSYLVANIA | 2020 |
| Autism spectrum disorder | Subnetwork-based Quantitative Imaging Biomarkers for Therapy Assessment in Autism | R01 | YALE UNIVERSITY | 2019 |
| Autism spectrum disorder | Prediction and Early Language Development in Young Children with ASD | R01 | UNIVERSITY OF WISCONSIN-MADISON | 2019 |
| Autism spectrum disorder | Investigating quantitative signatures of autism in toddlers | R01 | COLUMBIA UNIVERSITY TEACHERS COLLEGE | 2020 |
| Big data | Big Data Coursework for Computational Medicine | R25 | WEILL MEDICAL COLL OF CORNELL UNIV | 2016 |
| Big data | Fisk University/UIUC-Mayo KnowENG BD2K Center R25 Partnership | R25 | FISK UNIVERSITY | 2019 |
| Big data | Administrative Supplement Request for Transforming Analytical Learning in the Era of Big Data | R25 | UNIVERSITY OF MICHIGAN AT ANN ARBOR | 2016 |
| Big data | Developing Cloud-based tools for Big Neural Data | K01 | UNIVERSITY OF PENNSYLVANIA | 2015 |
| Big data | Summer Institute for Statistics of Big Data | R25 | UNIVERSITY OF WASHINGTON | 2016 |
| Breast cancer | Epidemiology of Molecular Risk Factors for Breast Cancer | R01 | VANDERBILT UNIVERSITY | 2011 |
| Breast cancer | HYPERMEDIA INTELLIGENT BREAST CANCER INFORMATION SYSTEM | R43 | SYUKHTUN RESEARCH | 1994 |
| Breast cancer | Integrative subtyping to improve therapeutic options for metastatic hormone receptor-positive breast cancer | K08 | STANFORD UNIVERSITY | 2020 |
| Breast cancer | Breast Cancer Risk Assessment with Bayesian Networks | R03 | BRIGHAM AND WOMEN'S HOSPITAL | 2003 |
| Breast cancer | Background parenchymal uptake (BPU) on molecular breast imaging as a novel breast cancer risk factor | R21 | MAYO CLINIC ROCHESTER | 2016 |
| Cardiovascular disease | Health Information Technology in Heart Failure Care | K08 | NEW YORK UNIVERSITY SCHOOL OF MEDICINE | 2017 |
| Cardiovascular disease | Advanced Heart Failure: Epidemiology and Outcomes | R01 | MAYO CLINIC ROCHESTER | 2020 |
| Cardiovascular disease | Dynamic prediction of heart failure using real-time functional status and EHR data in the ambulatory setting | K23 | UNIVERSITY OF CALIFORNIA, SAN FRANCISCO | 2020 |
| Cardiovascular disease | Integrated RF and B-mode Deformation Analysis for 4D Stress Echocardiography | R01 | YALE UNIVERSITY | 2016 |
| Cardiovascular disease | A PROGRAM FOR THE MANAGEMENT OF HEART FAILURE | R01 | MASSACHUSETTS INSTITUTE OF TECHNOLOGY | 1988 |
| Cell signaling pathways | Structure and Affinity: Computing FABP-Lipid Selectivity | R01 | JOHNS HOPKINS UNIVERSITY | 2006 |

© 2022 Eweje FR et al. *JAMA Network Open.*

| | | | | |
|---|---|---|---|---|
| Cell signaling pathways | Data-Driven Approaches for Molecular Docking | R01 | UNIVERSITY OF CALIFORNIA, SAN FRANCISCO | 2013 |
| Cell signaling pathways | Quantitative Modeling of Transcription Factor-DNA Binding | R35 | UNIVERSITY OF SOUTHERN CALIFORNIA | 2020 |
| Cell signaling pathways | Algorithmic identification of binding specificity mechanisms in proteins | R01 | LEHIGH UNIVERSITY | 2020 |
| Cell signaling pathways | Metal binding sites in macromolecular structures | R01 | UNIVERSITY OF VIRGINIA | 2019 |
| Centers for translational and computational research | Nathan Shock Center of Excellence in Basic Biology of Aging | P30 | UNIVERSITY OF WASHINGTON | 2020 |
| Centers for translational and computational research | Phase III COBRE: Multimodal Imaging of Neuropsychiatric Disorders (MIND) | P30 | THE MIND RESEARCH NETWORK | 2019 |
| Centers for translational and computational research | NIDA Center of Excellence OF Computational Drug Abuse Research (CDAR) | P30 | UNIVERSITY OF PITTSBURGH AT PITTSBURGH | 2018 |
| Centers for translational and computational research | Natural Product-Drug Interaction Research: The Roadmap to Best Practices | U54 | WASHINGTON STATE UNIVERSITY | 2018 |
| Centers for translational and computational research | Texas Gene Array Core | U01 | UNIVERSITY OF TEXAS, AUSTIN | 2010 |
| Clinically significant genetic variation | Comprehensive Characterization of Missense Mutants in Factor IX | R01 | BLOODWORKS | 2020 |
| Clinically significant genetic variation | Regulation of mRNA splicing by intronic genetic variants | R01 | INDIANA UNIV-PURDUE UNIV AT INDIANAPOLIS | 2018 |
| Clinically significant genetic variation | Discovering Novel Structural Genomic Rearrangements Using Deep Neural Networks | F31 | UNIVERSITY OF MICHIGAN AT ANN ARBOR | 2020 |
| Clinically significant genetic variation | Micropublications for Automating Genome Sequence Variant Interpretation from Medical Literature | R43 | GENOMENON, INC. | 2019 |
| Clinically significant genetic variation | Decrypting Variants of Uncertain Significance in Long-QT Syndrome | R01 | NORTHWESTERN UNIVERSITY AT CHICAGO | 2020 |
| Deep learning | Distributed Learning of Deep Learning Models for Cancer Research | U01 | STANFORD UNIVERSITY | 2020 |
| Deep learning | Learning to learn in structural biology with deep neural networks | R35 | UNIVERSITY OF ROCHESTER | 2020 |
| Deep learning | Deep LOGISMOS | R01 | UNIVERSITY OF IOWA | 2020 |
| Deep learning | Protected Radiomics Analysis Commons for Deep Learning in Biomedical Discovery | S10 | UNIVERSITY OF CHICAGO | 2018 |
| Deep learning | Can machines be trusted? Robustification of deep learning for medical imaging | R01 | UNIVERSITY OF WISCONSIN-MADISON | 2020 |
| Depression | Distributed networks underlying depression in epilepsy: a computational circuit-based approach to biomarker development | K23 | UNIVERSITY OF CALIFORNIA, SAN FRANCISCO | 2020 |
| Depression | An Expert System to Reduce Depression in Primary Care | R44 | PRO-CHANGE BEHAVIOR SYSTEMS, INC. | 2004 |
| Depression | Biosignatures of Treatment Remission in Major Depression | U01 | COLUMBIA UNIVERSITY HEALTH SCIENCES | 2015 |
| Depression | SPIMA: Signal Processing for Individualized Mood Assessment | R43 | DIMAGI, INC. | 2007 |
| Depression | Building Multistage Treatment Regimens for Depression after Acute Coronary SyndromeSyndrome | R21 | COLUMBIA UNIVERSITY HEALTH SCIENCES | 2017 |
| Diabetes | Detecting, Understanding, and Reducing Diabetes Belt Preventive Care Disparities | R01 | UNIVERSITY OF VIRGINIA | 2020 |

© 2022 Eweje FR et al. *JAMA Network Open.*

| | | | | |
|---|---|---|---|---|
| Diabetes | Validation of Survey Questions to Distinguish Type 1 and Type 2 Diabetes Among Adults With Diabetes | U01 | WESTAT, INC. | 2018 |
| Diabetes | Southern California Clinical Center of the Type 1 Diabetes in Acute Pancreatitis Consortium | U01 | CEDARS-SINAI MEDICAL CENTER | 2020 |
| Diabetes | Clinical, Radiologic and Biochemical Factors Related to Diabetes Development after Acute Pancreatitis | U01 | JOHNS HOPKINS UNIVERSITY | 2020 |
| Diabetes | Multi-institutional Consortium for CER in Diabetes Treatment and Prevention | R01 | KAISER FOUNDATION RESEARCH INSTITUTE | 2010 |
| Drug discovery | Predictive Guidelines for Penetrance and Discovery of Broad-Spectrum Antibiotics | R01 | UNIVERSITY OF ILLINOIS AT URBANA-CHAMPAIGN | 2019 |
| Drug discovery | Rules for penetrating the Gram-negative bacterial envelope | R56 | UNIVERSITY OF KENTUCKY | 2018 |
| Drug discovery | West Coast Metabolomics Center for Compound Identification | U2C | UNIVERSITY OF CALIFORNIA AT DAVIS | 2020 |
| Drug discovery | MegaTrans ‚Äì human transporter machine learning models | R41 | COLLABORATIONS PHARMACEUTICALS, INC. | 2019 |
| Drug discovery | Development of a Biocatalytic Platform for Convergent Synthesis of Chiral Amines | R01 | CALIFORNIA INSTITUTE OF TECHNOLOGY | 2020 |
| EEG | Epileptic Seizures in the Neonatal EEG | R01 | UNIVERSITY OF HOUSTON | 2004 |
| EEG | Wireless EEG/PSG System with Novel Artifact Removal | R43 | CLEVELAND MEDICAL DEVICES, INC. | 2004 |
| EEG | MULTICHANNEL EEG DATA COMPRESSION | R01 | UNIVERSITY OF PITTSBURGH AT PITTSBURGH | 2001 |
| EEG | CLUSTERING AND HYPER-LINKING OF LONG-TERM EEGS | R44 | PERSYST DEVELOPMENT CORPORATION | 2000 |
| EEG | Automatic discovery and processing of EEG cohorts from clinical records | U01 | TEMPLE UNIV OF THE COMMONWEALTH | 2017 |
| Electronic health record | National Infrastructure for Standardized and Portable EHR Phenotyping Algorithms | R01 | WEILL MEDICAL COLL OF CORNELL UNIV | 2019 |
| Electronic health record | From enrichment to insights | R01 | STANFORD UNIVERSITY | 2020 |
| Electronic health record | Algorithms to Identify Systemic Lupus from Electronic Health Record Data | R21 | NORTHWESTERN UNIVERSITY AT CHICAGO | 2018 |
| Electronic health record | Integrating EHR and Genomics to Predict Multiple Sclerosis Drug Response | R01 | UNIVERSITY OF PITTSBURGH AT PITTSBURGH | 2020 |
| Electronic health record | Biases introduced by filtering electronic health records for patients with "complete data" | R01 | HARVARD MEDICAL SCHOOL | 2020 |
| Environmental health | Cardiovascular health and exposure to PM2.5 constituents: a multi-cohort study | R01 | EMORY UNIVERSITY | 2020 |
| Environmental health | Environment, Metabolomics, and PD | R21 | UNIVERSITY OF CALIFORNIA LOS ANGELES | 2020 |
| Environmental health | Biomarkers and Genes Associated with Placental Development and Function in Response to Environmental Pollution | R01 | UNIVERSITY OF CALIFORNIA LOS ANGELES | 2020 |
| Environmental health | The Impact of Air Pollution Exposure on COVID-19 Severity and Mortality | R01 | KAISER FOUNDATION RESEARCH INSTITUTE | 2020 |
| Environmental health | Prenatal Exposure to Pesticide Mixtures and Childhood ADHD | R00 | UNIVERSITY OF ARIZONA | 2020 |
| Familial genetics | METHODS FOR FUNCTIONAL/COMPARATIVE GENOMICS | R01 | IOWA STATE UNIVERSITY | 2003 |
| Familial genetics | NIDA STRENGTHENING WASHINGTON, DC FAMILIES GRANT | R01 | UNIVERSITY OF UTAH | 2004 |

© 2022 Eweje FR et al. *JAMA Network Open.*

| | | | | |
|---|---|---|---|---|
| Familial genetics | Linking Information, Families and Technology (LIFT) | R43 | KIT SOLUTIONS, INC. | 2005 |
| Familial genetics | The Electronic Medical Records and Genomics (eMERGE) Network, Phase III | U01 | KAISER FOUNDATION RESEARCH INSTITUTE | 2019 |
| Familial genetics | Strengthening Families Program Online Web Version for Teens | R43 | STRENGTHENING FAMILIES PROGRAM, LLC | 2018 |
| Functional mutations | Tracing the evolution of the human mutation rate | F32 | STANFORD UNIVERSITY | 2017 |
| Functional mutations | A statistical framework to systematically characterize cancer driver mutations in noncoding genomic regions | R21 | DANA-FARBER CANCER INST | 2020 |
| Functional mutations | Modeling of Protein Complexes and Missense Mutations | R01 | RESEARCH INST OF FOX CHASE CAN CTR | 2009 |
| Functional mutations | Massively parallel functional analyses of human PTEN variants | F31 | OREGON HEALTH & SCIENCE UNIVERSITY | 2019 |
| Functional mutations | Informatics Tools for High-throughput Analysis of Cancer Mutations | U01 | JOHNS HOPKINS UNIVERSITY | 2015 |
| Gene mapping | INTELLIGENT AUTOMATED RESTRICTION MAPPING TOOL | R01 | UNIVERSITY OF ILLINOIS AT CHICAGO | 1995 |
| Gene mapping | Methods for Human Genetic Mapping | R01 | UNIVERSITY OF CHICAGO | 2012 |
| Gene mapping | Enhanced Gene Identification in Complex Traits Using Kernel Machines | R01 | EMORY UNIVERSITY | 2015 |
| Gene mapping | LLSF MAPPING FOR INDEXING AND RETRIEVAL OF MEDLINE | R29 | CARNEGIE-MELLON UNIVERSITY | 1999 |
| Gene mapping | Functional Data Analysis of Longitudinally Measured Genetic Traits. | R03 | COLUMBIA UNIVERSITY HEALTH SCIENCES | 2010 |
| HIV | Big Data analytics of HIV treatment gaps in South Carolina: Identification and prediction | R01 | UNIVERSITY OF SOUTH CAROLINA AT COLUMBIA | 2020 |
| HIV | Developing an artificial intelligence-based mHealth intervention to increase HIV testing in Malaysia | R21 | YALE UNIVERSITY | 2020 |
| HIV | Determinants of voluntary HIV testing among inmates | F30 | UNIV OF NORTH CAROLINA CHAPEL HILL | 2009 |
| HIV | Developing an Artificial Intelligence Chatbot to Promote HIV Testing | R21 | YALE UNIVERSITY | 2020 |
| HIV | Addressing Major HIV Prevention and Health Outcomes Questions in an Era of Universal ART: Mentoring in a Community-Randomized Trial | K24 | BRIGHAM AND WOMEN'S HOSPITAL | 2019 |
| Immunology | Machine Learning for Integrative Modeling of the Immune System in Clinical Settings | R35 | STANFORD UNIVERSITY | 2020 |
| Immunology | SIMULATION MODELING OF THE IMMUNE SYSTEM | R43 | D.D.G., INC. | 1988 |
| Immunology | Immune cells and STAT3-mediated inflammation in calcific aortic valve disease | F30 | VANDERBILT UNIVERSITY | 2020 |
| Immunology | Systems-level identification of key regulators deciding immune cell state | R01 | UNIVERSITY OF CALIFORNIA, SAN DIEGO | 2020 |
| Immunology | Comprehensive analysis of human adaptive immune receptors to elucidate correlates of Epstein-Barr virus disease suppression | DP5 | UNIVERSITY OF KANSAS LAWRENCE | 2020 |
| Intelligent search engines and data visualization | Continued Maintenance and Development of Software: Integrated Genome Browser and | R01 | UNIVERSITY OF NORTH CAROLINA CHARLOTTE | 2012 |
| Intelligent search engines and data visualization | INTELLIGENT BIOMEDICAL ASSISTANT | R01 | UNIVERSITY OF PITTSBURGH AT PITTSBURGH | 1991 |

© 2022 Eweje FR et al. *JAMA Network Open.*

| | | | | |
|---|---|---|---|---|
| Intelligent search engines and data visualization | Visual Data Extraction and Conversion Programming Tool | R33 | IOWA STATE UNIVERSITY | 2005 |
| Intelligent search engines and data visualization | Evidence Extraction Systems for the Molecular Interaction Literature | R01 | UNIVERSITY OF SOUTHERN CALIFORNIA | 2020 |
| Intelligent search engines and data visualization | Bayesian Statistics and Algorithms for Homology Modeling | R01 | INSTITUTE FOR CANCER RESEARCH | 2006 |
| Interpersonal communication technologies | TOPIC PREDICTION FOR AUGMENTATIVE COMMUNICATION SYSTEMS | R43 | INVOTEK, INC. | 1999 |
| Interpersonal communication technologies | Automated Coding of eCoaching Exchanges to Promote Healthier Eating | R21 | WAYNE STATE UNIVERSITY | 2017 |
| Interpersonal communication technologies | Communication Ability and Work Re-Entry in Adults with Brain Injury | F31 | UNIVERSITY OF WISCONSIN-MADISON | 2012 |
| Interpersonal communication technologies | Translational refinement of adaptive communication system for locked-in patients | R01 | OREGON HEALTH & SCIENCE UNIVERSITY | 2013 |
| Interpersonal communication technologies | Ethical Considerations for Language Modeling within Brain-Computer Interfaces | R01 | OREGON HEALTH & SCIENCE UNIVERSITY | 2019 |
| Kidney disease | Novel Pathways for Kidney Stone Formation | R01 | BRIGHAM AND WOMEN'S HOSPITAL | 2020 |
| Kidney disease | Developing and validating prognostic metabolomic signatures of diabetic kidney disease | R01 | UNIVERSITY OF CALIFORNIA, SAN DIEGO | 2020 |
| Kidney disease | Promoting Utilization of Kidneys by Improving Patient Level Decision Making | R21 | NORTHWESTERN UNIVERSITY | 2017 |
| Kidney disease | The comparative effectiveness of kidney transplantation in advanced CKD | K08 | JOHNS HOPKINS UNIVERSITY | 2016 |
| Kidney disease | Prediction of Major Adverse Kidney Events and Recovery (Pred-MAKER) in COVID-19 Patients | R01 | ICAHN SCHOOL OF MEDICINE AT MOUNT SINAI | 2020 |
| Knowledge bases | DEVELOPING QMR KNOWLEDGE BASE INTO A RESOURCE | R01 | UNIVERSITY OF PITTSBURGH AT PITTSBURGH | 1993 |
| Knowledge bases | CANCER PROTEIN DATA BASE | R43 | LARGE SCALE BIOLOGY CORPORATION | 1986 |
| Knowledge bases | CLINICAL DECISION SYSTEMS RESEARCH RESOURCE | R24 | UNIVERSITY OF PITTSBURGH AT PITTSBURGH | 1989 |
| Knowledge bases | CADUCEUS: A COMPUTER BASED DIAGNOSTIC CONSULTANT | R01 | UNIVERSITY OF PITTSBURGH AT PITTSBURGH | 1987 |
| Knowledge bases | ENHANCED FOOD COMPOSITION RESEARCH DATA BASE | N43 | SOHAR, INC. | 1989 |
| Knowledge representation and reasoning | CRCNS: Representational foundations of adaptive behavior in natural and artificial | R01 | PRINCETON UNIVERSITY | 2017 |
| Knowledge representation and reasoning | The Developmental Antecedents and Cognitive Correlates of Script-like Attachment | F32 | UNIVERSITY OF MINNESOTA | 2015 |
| Knowledge representation and reasoning | DIGITAL IMAGE REPRESENTATIONS FOR TOMOGRAPHIC RADIOLOGY | R01 | UNIVERSITY OF PENNSYLVANIA | 1998 |
| Knowledge representation and reasoning | Neural signatures of learning complex environments in the amygdala-prefrontal network | K99 | COLUMBIA UNIVERSITY HEALTH SCIENCES | 2020 |
| Knowledge representation and reasoning | Neural representation of the geometry and functionality in a scene | R01 | JOHNS HOPKINS UNIVERSITY | 2020 |
| Language development and reading comprehension | PREDICTING OUTCOME IN CHILDREN WITH NEW-ONSET TICS USING NEUROIMAGING DATA | R21 | WASHINGTON UNIVERSITY | 2016 |
| Language development and | SOFTWARE INTERVENTION FOR ADVANCED SYNTAX | R43 | LAUREATE LEARNING SYSTEMS, INC. | 1999 |

© 2022 Eweje FR et al. *JAMA Network Open.*

| | | | | |
|---|---|---|---|---|
| reading comprehension | DEVELOPMENT | | | |
| Language development and reading comprehension | Cognitive Representation in Specific Language Impairment | R01 | SAN DIEGO STATE UNIVERSITY | 2009 |
| Language development and reading comprehension | Predicting Heterogeneous Neurodevelopmental Outcomes in School-age Children with Early Caregiving Adversities | R01 | COLUMBIA UNIV NEW YORK MORNINGSIDE | 2020 |
| Language development and reading comprehension | Verb learning and the early development of sentence comprehension | R01 | UNIVERSITY OF ILLINOIS AT URBANA-CHAMPAIGN | 2018 |
| Literature review | Hybrid Approaches to Optimizing Evidence Synthesis via Machine Learning and Crowdsourcing | R03 | NORTHEASTERN UNIVERSITY | 2016 |
| Literature review | Text Mining Pipeline to Accelerate Systematic Reviews in Evidence-Based Medicine | R01 | UNIVERSITY OF ILLINOIS AT CHICAGO | 2019 |
| Literature review | Developing Methods to Improve Systematic Reviews Using Clinical Trial Registries | R03 | BOSTON CHILDREN'S HOSPITAL | 2016 |
| Literature review | Screening Nonrandomized Studies for Inclusion in Systematic Reviews of Evidence | R00 | UNIVERSITY OF PITTSBURGH AT PITTSBURGH | 2012 |
| Literature review | SWIFT-ActiveScreener: research and development of an intelligent web-based document screening system | R43 | SCIOME, LLC | 2017 |
| Liver disease | Hepatic Steatosis and the Lipid Metabolome | R01 | UNIVERSITY OF TEXAS MED BR GALVESTON | 2009 |
| Liver disease | Development of a Machine Learning Model for Liver Transplantation | F32 | JOHNS HOPKINS UNIVERSITY | 2020 |
| Liver disease | HEPATOCYTE-DERIVED MIF: A KEY CONTRIBUTOR TO ALCOHOLIC LIVER DISEASE | R00 | UNIVERSITY OF TEXAS HLTH SCI CTR HOUSTON | 2020 |
| Liver disease | Development of a Machine Learning Model to Integrate Clinical, Laboratory, Sonographic, and Elastographic Data for Noninvasive Liver Tissue Characterization in NAFLD | R01 | MASSACHUSETTS GENERAL HOSPITAL | 2020 |
| Liver disease | NOVEL STRATEGIES TO IMPROVE LIVER CANCER SURVEILLANCE UPTAKE AND EARLY DETECTION | U01 | UNIVERSITY OF MICHIGAN AT ANN ARBOR | 2020 |
| Lung cancer and COPD | EARLY DETECTION OF LUNG CANCER USING METABOLOMIC LIPID PROFILING | R21 | RUSH UNIVERSITY MEDICAL CENTER | 2014 |
| Lung cancer and COPD | FETAL LUNG BIOCHEMISTRY AND 3-DIMENSIONAL MICROANATOMY | R01 | DUKE UNIVERSITY | 1991 |
| Lung cancer and COPD | Lung cancer in East Africa and the relationship to HIV-1 infection: epidemiology, molecular characterization and imaging | U54 | CASE WESTERN RESERVE UNIVERSITY | 2020 |
| Lung cancer and COPD | Development of a Novel Lung Function Imaging Modality for comprehensive management of lung cancer | R01 | UNIVERSITY OF COLORADO DENVER | 2020 |
| Lung cancer and COPD | Histopathology correlated quantitative analysis of lung nodules with LDCT for early detection of lung cancer | U01 | UNIVERSITY OF MICHIGAN AT ANN ARBOR | 2020 |
| Mass spectroscopy | Machine learning analysis of tandem mass spectra | R01 | UNIVERSITY OF WASHINGTON | 2012 |
| Mass spectroscopy | Self Correcting Nanoflow LC-MS for Clinical Proteomics | R01 | UNIVERSITY OF WASHINGTON | 2016 |
| Mass spectroscopy | Proteomics processing using networked instrument router* | R43 | USERSPACE CORPORATION | 2005 |
| Mass spectroscopy | Proteomics processing using networked instrument router | R43 | USERSPACE CORPORATION | 2004 |
| Mass spectroscopy | Optimization and joint modeling for peptide detection by tandem mass spectrometry | R01 | UNIVERSITY OF WASHINGTON | 2020 |
| Memory | Neural mechanisms for reducing interference during episodic memory formation | R01 | UNIVERSITY OF OREGON | 2015 |

© 2022 Eweje FR et al. *JAMA Network Open.*

| | | | | |
|---|---|---|---|---|
| Memory | The role of memory reactivation in emotional memory suppression and regulation | F99 | PRINCETON UNIVERSITY | 2020 |
| Memory | Investigating Cognitive and Neural Bases of Working Memory | R01 | UNIVERSITY OF WISCONSIN-MADISON | 2013 |
| Memory | The effects of film music on neural activity in higher-order brain areas and comprehension for the film narrative | F99 | PRINCETON UNIVERSITY | 2020 |
| Memory | Testing a Model of Competitive Memory Retrieval | F31 | PRINCETON UNIVERSITY | 2006 |
| Metabolic syndrome and metabolic processes | Pathway Prediction and Assessment Integrating Multiple Evidence Types | R01 | SRI INTERNATIONAL | 2009 |
| Metabolic syndrome and metabolic processes | Computational Annotation of Orphan Metabolic Activities | R01 | COLUMBIA UNIVERSITY HEALTH SCIENCES | 2009 |
| Metabolic syndrome and metabolic processes | Dietary modulation of gut microbiome and host gene expression across human evolution and the emergence of modern human disease | R01 | J. CRAIG VENTER INSTITUTE, INC. | 2019 |
| Metabolic syndrome and metabolic processes | Constitutional and metabolic factors associated with the development of Hand OA | R01 | TUFTS MEDICAL CENTER | 2018 |
| Metabolic syndrome and metabolic processes | System-wide Study of Transcriptional Control of Metabolism | R21 | LOS ALAMOS NAT SECTY-LOS ALAMOS NAT LAB | 2008 |
| Molecular genetics | DNA Polymerase Database | R44 | NEW ENGLAND BIOLABS, INC. | 2010 |
| Molecular genetics | Molecular mechanisms of germline DNA repair and DNA damage response | R01 | HARVARD MEDICAL SCHOOL | 2016 |
| Molecular genetics | DNA 3.0: Developing novel enzymes for DNA synthesis with deep learning and combinatorial genetics | R43 | PRIMORDIAL GENETICS, INC | 2020 |
| Molecular genetics | Comprehensive breakpoint analyses for simultaneous quantification of all DNA double strand break repair pathways | R33 | SLOAN-KETTERING INST CAN RESEARCH | 2020 |
| Molecular genetics | STATISTICAL STUDIES OF DNA EVOLUTION | R37 | UNIVERSITY OF CHICAGO | 2009 |
| Motion tracking and artifact reduction | Machine Learning and Deformable Model-based 4D Characterization of Cardiac Dyssynchrony from MRI | R01 | RUTGERS, THE STATE UNIV OF N.J. | 2020 |
| Motion tracking and artifact reduction | Systems Biology Analysis of Cardiac Electrical Activity and Arrhythmias. | R01 | UNIVERSITY OF WASHINGTON | 2020 |
| Motion tracking and artifact reduction | Cloud-based High-throughput Acquisition and Analytics of Zebrafish Electrocardiogram for Cardiac Studies and Drug Development | R44 | SENSORIIS, INC. | 2020 |
| Motion tracking and artifact reduction | Innovative MRI-based Characterization of Cardiac Dyssynchrony | R01 | RUTGERS, THE STATE UNIV OF N.J. | 2018 |
| Motion tracking and artifact reduction | SIGNAL PROCESSING/AUTOMATION FOR CARDIAC OUTPUT MONITOR | R43 | AXON MEDICAL, INC. | 1994 |
| Motor function | Spinal Epidural Electrode Array To Facilitate Standing and Stepping After SCI | U01 | UNIVERSITY OF CALIFORNIA LOS ANGELES | 2018 |
| Motor function | Enabling forelimb function with agonist drug and epidural stimulation in SCI | U01 | UNIVERSITY OF CALIFORNIA LOS ANGELES | 2017 |
| Motor function | Data Mining to Identify Motor Fluctuations in PD | R21 | SPAULDING REHABILITATION HOSPITAL | 2006 |
| Motor function | MECHANISMS OF DEEP BRAIN STIMULATION | R01 | WASHINGTON UNIVERSITY | 2015 |
| Motor function | Reinforcement Learning for Closed-Loop Deep Brain Stimulation | F31 | UNIVERSITY OF MINNESOTA | 2019 |
| Mouse modeling | Chemoreceptor Cell Development in the Carotid Body | R01 | JOHNS HOPKINS UNIVERSITY | 2009 |

© 2022 Eweje FR et al. *JAMA Network Open.*

| | | | | |
|---|---|---|---|---|
| Mouse modeling | Towards automated phenotyping in epilepsy | R21 | STANFORD UNIVERSITY | 2018 |
| Mouse modeling | Development of mosaic mouse models of HCC for genetic interspecies inference | R00 | RESEARCH INST OF FOX CHASE CAN CTR | 2019 |
| Mouse modeling | Mining Genomic Data in FaceBase for Cleft Genes | R03 | UNIVERSITY OF TEXAS HLTH SCI CTR HOUSTON | 2019 |
| Mouse modeling | A Novel Modifier Locus Suppressing ApcMin Intestinal Polyposis | F31 | THOMAS JEFFERSON UNIVERSITY | 2009 |
| Natural language processing | Natural language processing for clinical and translational research | R01 | MAYO CLINIC ROCHESTER | 2016 |
| Natural language processing | Open Health Natural Language Processing Collaboratory | U01 | MAYO CLINIC ROCHESTER | 2020 |
| Natural language processing | A Biomedical Natural Language Processing Resource | R01 | COLUMBIA UNIVERSITY HEALTH SCIENCES | 2008 |
| Natural language processing | Annotation, development and evaluation for clinical information extraction | R01 | UNIVERSITY OF CALIFORNIA, SAN DIEGO | 2013 |
| Natural language processing | Annotation, development and evaluation for clinical information extraction (transfer) | R01 | BOSTON CHILDREN'S HOSPITAL | 2013 |
| Neural circuits | Image-based modeling of functional connectivity in neural networks at single-cell resolution | K25 | UT SOUTHWESTERN MEDICAL CENTER | 2020 |
| Neural circuits | Experimental examinations of the mechanisms that generate the responses of midbra | R01 | HARVARD UNIVERSITY | 2017 |
| Neural circuits | Uncovering Population-Level Cellular Relationships to Behavior via Mesoscale Networks | R01 | DUKE UNIVERSITY | 2019 |
| Neural circuits | Transcriptome-based systematic discovery of GABAergic neurons in the neocortex | R01 | COLD SPRING HARBOR LABORATORY | 2020 |
| Neural circuits | Crowd coding in the brain:3D imaging and control of collective neuronal dynamics | U01 | UNIV OF MARYLAND, COLLEGE PARK | 2016 |
| Object tracking and recognition | AN INTERACTIVE BIOMEDICAL IMAGE PROCESSOR/ANALYZER | R44 | AMERICAN INNOVISION, INC. | 1986 |
| Object tracking and recognition | Transmission of Information in the Visual System | R01 | NEW YORK UNIVERSITY | 2009 |
| Object tracking and recognition | Closing the loop on markerless object tracking | R03 | BROWN UNIVERSITY | 2020 |
| Object tracking and recognition | ENCODING REACH AND GRASP IN CEREBELLAR NEURONAL ACTIVITY | F32 | UNIVERSITY OF MINNESOTA TWIN CITIES | 1999 |
| Object tracking and recognition | Understanding the circuit for topological object tracking | DP1 | CALIFORNIA INSTITUTE OF TECHNOLOGY | 2016 |
| Older adults | Cognitive and social aspects of decision-making | R15 | UNIVERSITY OF COLORADO AT COLORADO SPGS | 2002 |
| Older adults | Web-based App to Alleviate Loneliness and Isolation in Older Adults at Home | R43 | VIGOROUS MIND, INC. | 2018 |
| Older adults | Frailty and Risk Prediction in Older Adults Considering Kidney Transplantation | R01 | JOHNS HOPKINS UNIVERSITY | 2017 |
| Older adults | Frailty and Risk Prediction in Older Adults Considering Kidney Transplantation Supp Olorundare | R01 | JOHNS HOPKINS UNIVERSITY | 2015 |
| Older adults | Statistical methods for vitamin D targets for functional outcomes in older adults | R01 | UNIVERSITY OF MARYLAND BALTIMORE | 2016 |
| Ontology generation | Protege: A Knowledge-Engineering Environment for Advancing Biomedical Sciences | R01 | STANFORD UNIVERSITY | 2019 |

© 2022 Eweje FR et al. *JAMA Network Open.*

| | | | | |
|---|---|---|---|---|
| Ontology generation | Services to support the OBO foundry standards | R24 | LA JOLLA INSTITUTE FOR IMMUNOLOGY | 2019 |
| Ontology generation | Protege: An Ontology-Development Platform for Biomedical Scientists | R01 | STANFORD UNIVERSITY | 2016 |
| Ontology generation | The ODIE Toolkit - Software for Information Extraction and Biomedical Ontology De | R01 | UNIVERSITY OF PITTSBURGH AT PITTSBURGH | 2010 |
| Ontology generation | Collaborative Development of Biomedical Ontologies and Terminologies | R01 | STANFORD UNIVERSITY | 2010 |
| Other cancer | Predicting brain tumor progression via multiparametric image analysis and modelin | R01 | UNIVERSITY OF PENNSYLVANIA | 2018 |
| Other cancer | Systems Microscopy Analysis of Tumor Cell Motility in Microenvironment Context | R00 | TEMPLE UNIV OF THE COMMONWEALTH | 2017 |
| Other cancer | Molecular Characterization of Parotid Gland Tumors | R56 | UNIVERSITY OF CALIFORNIA LOS ANGELES | 2012 |
| Other cancer | Molecular interactions and restoration strategies of PTEN and p53 in gliomas | R01 | UNIVERSITY OF VIRGINIA | 2014 |
| Other cancer | COMPUTING TUMOR DETECTION PERFORMANCE IN MEDICAL IMAGING | K01 | UNIVERSITY OF ARIZONA | 2004 |
| Other chemical compound characterization | SYSTEM FOR MACHINE READING OF CHEMICAL SYMBOLS | R43 | WESTERN RESEARCH COMPANY, INC. | 1988 |
| Other chemical compound characterization | INTELLIGENT SOFTWARE FOR CHEMICAL DIAGRAMS | R44 | COMPUTER HUMAN INTERFACE, INC. | 1990 |
| Other chemical compound characterization | KNOWLEDGE-BASED CHEMICAL SYMBOL RECOGNITION SYSTEM | R43 | WESTERN RESEARCH COMPANY, INC. | 1993 |
| Other chemical compound characterization | Digital representation of chemical mixtures to aid drug discovery and formulation | R44 | COLLABORATIVE DRUG DISCOVERY, INC. | 2020 |
| Other chemical compound characterization | MCASE QSAR Expert System for Salmonella Mutagenicity | R44 | MULTICASE, INC. | 2003 |
| Other child development | When cues converge: multiple regularities in language acquisition | F31 | UNIVERSITY OF WISCONSIN-MADISON | 2008 |
| Other child development | Infant statistical learning: Resilience, longevity, and specificity | R01 | UNIVERSITY OF TENNESSEE KNOXVILLE | 2019 |
| Other child development | Continued Development of Infant Brain Analysis Tools | R01 | UNIV OF NORTH CAROLINA CHAPEL HILL | 2020 |
| Other child development | Social and Statistical Mechanisms of Prelinguistic Vocal Learning | R03 | CORNELL UNIVERSITY | 2010 |
| Other child development | Linking Statistical Learning to Vocabulary Development | R03 | UNIVERSITY OF CALIFORNIA AT DAVIS | 2011 |
| Other dementia | Dementia Risk Prediction Pooling Project | R61 | NORTHWESTERN UNIVERSITY AT CHICAGO | 2020 |
| Other dementia | Identifying Mechanisms of Dementia:  Role for MRI in the Era of Molecular Imaging | R01 | MAYO CLINIC ROCHESTER | 2010 |
| Other dementia | Changing Talk Online (CHATO): A Pragmatic Trial to Reduce Behavioral Symptoms in Dementia Care | R61 | UNIVERSITY OF KANSAS MEDICAL CENTER | 2019 |
| Other dementia | CATcare: Cognitive Assistive Technology for Dementia Homecare | R15 | OKLAHOMA STATE UNIVERSITY STILLWATER | 2019 |
| Other dementia | Social Assistive Robot Interface for People with Alzheimer's and Other Dementias to Aid in Care Management | R44 | ADVANCED MEDICAL ELECTRONICS CORPORATION | 2019 |

© 2022 Eweje FR et al. *JAMA Network Open.*

| | | | | |
|---|---|---|---|---|
| Other infectious disease | Nosocomial infections: Automated typing and data mining | R43 | MICROBIAL ID, INC. | 2001 |
| Other infectious disease | Seroepidemiologic methods to identify hotspots of trachoma and predict future infection | R03 | UNIVERSITY OF CALIFORNIA, SAN FRANCISCO | 2020 |
| Other infectious disease | Seroepidemiologic methods to identify hotspots of trachoma and predict future infection | R03 | UNIVERSITY OF CALIFORNIA BERKELEY | 2019 |
| Other infectious disease | ANALYSIS OF PROBLEMS IN OCULAR INFLAMMATORY DISEASE | R01 | UNIVERSITY OF CALIFORNIA SAN FRANCISCO | 1985 |
| Other infectious disease | Identifying Risk Factors for Antibiotic Resistance via Integration of Epidemiology and Metagenomics | K01 | UNIVERSITY OF TX MD ANDERSON CAN CTR | 2020 |
| Other mental health | Estimating Population Effects in Mental Health Research Using Meta-Analysis | R00 | DUKE UNIVERSITY | 2020 |
| Other mental health | Discovery of Mental Health and Inflammation (MHAIN) Interactome | R01 | UNIVERSITY OF PITTSBURGH AT PITTSBURGH | 2014 |
| Other mental health | Assisted Identification and Navigation of Early Mental Health Symptoms in Youth | R56 | KAISER FOUNDATION RESEARCH INSTITUTE | 2018 |
| Other mental health | CBTpro: Scaling up CBT for psychosis using simulated patients and spoken language technologies | R42 | LYSSN.IO, INC. | 2020 |
| Other mental health | Impact of Telemedicine on Medicare Beneficiaries with Mental Illness | R01 | HARVARD MEDICAL SCHOOL | 2020 |
| Other patient safety | Reducing Drug Name Confusion With Better Search Software | R44 | PHARM I.R. | 2008 |
| Other patient safety | MODEL MISSPECIFICATION DETECTION IN DIPOLE ANALYSIS | R43 | ABRATECH CORPORATION | 1994 |
| Other patient safety | Utility of Predictive Systems to identify Inpatient Diagnostic Errors: The UPSIDE Study | R01 | UNIVERSITY OF CALIFORNIA, SAN FRANCISCO | 2020 |
| Other patient safety | Application of a Machine Learning to Enhance e-Triggers to Detect and Learn from Diagnostic Safety Events | R01 | BAYLOR COLLEGE OF MEDICINE | 2020 |
| Other patient safety | NLP to Improve Accuracy and Quality of Dictated Medical Documents | R01 | BRIGHAM AND WOMEN'S HOSPITAL | 2017 |
| Pain | Evaluation of Pain in Chronic Pancreatitis using the NAPS2 cohorts | R21 | UNIVERSITY OF PITTSBURGH AT PITTSBURGH | 2015 |
| Pain | CROSSCULTURAL STUDY OF PAIN--PATIENTS AND DENTISTS | R29 | UNIVERSITY OF WASHINGTON | 1996 |
| Pain | Autonomous Pain Recognition in Non-Verbal and Critically Ill Patients | R21 | UNIVERSITY OF FLORIDA | 2020 |
| Pain | Molecular Epidemiology of Neuropathic Pain in Head and Neck Cancer | R01 | UNIVERSITY OF TX MD ANDERSON CAN CTR | 2015 |
| Pain | fMRI-based Biomarkers for Multiple Components of Pain | R01 | UNIVERSITY OF COLORADO | 2017 |
| Pediatrics | Pediatric sepsis prediction: a machine learning solution for patient diversity | R43 | DASCENA, INC. | 2018 |
| Pediatrics | Molecular Genetic Investigation of Pediatric Myelodysplastic Syndrome | R24 | BOSTON CHILDREN'S HOSPITAL | 2012 |
| Pediatrics | Rule-based Semantics and Big Data Based Methods for Effective Clinical Decision Support (CDS): A Pediatric Severe Sepsis Case Study using ICU Data | R43 | COMPUTER TECHNOLOGY ASSOCIATES, INC. | 2017 |
| Pediatrics | Enhancing Quality in Pediatrics Sepsis with Shock Prediction and Early Electronic Decision Support (EQUIP with SPEED) | K08 | UNIVERSITY OF COLORADO DENVER | 2020 |
| Pediatrics | Automatic Rib Fracture Detection in Pediatric Radiography to | R21 | MICHIGAN STATE UNIVERSITY | 2020 |

© 2022 Eweje FR et al. *JAMA Network Open.*

| | | | | |
|---|---|---|---|---|
| | Identify Non-Accidental Trauma | | | |
| Population genetics | Database of Functional SNPs in Cancer-Related Environmentally Responsive Genes | R01 | YALE UNIVERSITY | 2009 |
| Population genetics | Genome-Wide Association Analysis of Bladder Cancer | U01 | UNIVERSITY OF TX MD ANDERSON CAN CTR | 2012 |
| Population genetics | Analysis of the Functional Impact of Coding region SNPs | R01 | UNIV OF MARYLAND, COLLEGE PARK | 2010 |
| Population genetics | Machine learning to identify predictive SNPs and complex interaction effects | R21 | VIRGINIA POLYTECHNIC INST AND ST UNIV | 2010 |
| Population genetics | Molecular Genetics of HNPCC | R01 | UNIVERSITY OF TX MD ANDERSON CAN CTR | 2011 |
| Population health screening | Improving Critical Congenital Heart Disease Screening and Detection of "Secondary" Targets | R21 | UNIVERSITY OF CALIFORNIA AT DAVIS | 2020 |
| Population health screening | New approaches to optimizing the application and measuring the impact of community-based tuberculosis interventions | DP2 | BRIGHAM AND WOMEN'S HOSPITAL | 2019 |
| Population health screening | Familial hypercholesterolemia screening in children: population impact of phenotype, genotype, and cascade approaches | R01 | COLUMBIA UNIVERSITY HEALTH SCIENCES | 2020 |
| Population health screening | Studying Colorectal Cancer Effectiveness of Screening Strategies (SuCCESS) | U54 | KAISER FOUNDATION HEALTH PLAN OF WASHINGTON | 2014 |
| Population health screening | Identifying Receipt of Colorectal Cancer Screening | R21 | VANDERBILT UNIVERSITY | 2008 |
| Prostate cancer | Prostate Cancer Detection by Serum Proteomic Profiling | R03 | UNIVERSITY OF CALIFORNIA IRVINE | 2005 |
| Prostate cancer | Genetic & epigenetic analysis of angiogenesis genes in recurrent prostate cancer | R01 | H. LEE MOFFITT CANCER CTR & RES INST | 2012 |
| Prostate cancer | Gene-Gene Interactions and Their Functional Roles in Prostate Cancer Aggressiveness | R21 | LSU HEALTH SCIENCES CENTER | 2017 |
| Prostate cancer | MRI Imaging and Biomarkers for Early Detection of Aggressive Prostate Cancer | U01 | UNIVERSITY OF MIAMI SCHOOL OF MEDICINE | 2020 |
| Prostate cancer | A Serum Marker for Aggressive Prostate Cancer | R01 | STANFORD UNIVERSITY | 2010 |
| Protein structure and binding prediction | Protein structural disorder and ubiquitination | R21 | ROCKEFELLER UNIVERSITY | 2008 |
| Protein structure and binding prediction | Morphometry Biomedical Informatics Research Network | U24 | MASSACHUSETTS GENERAL HOSPITAL | 2008 |
| Protein structure and binding prediction | Exon recognition during constitutive pre-mRNA splicing | R01 | COLUMBIA UNIV NEW YORK MORNINGSIDE | 2008 |
| Protein structure and binding prediction | BECKON - Block Estimate Chain: creating Knowledge ON demand & protecting privacy | R00 | UNIVERSITY OF CALIFORNIA, SAN DIEGO | 2020 |
| Protein structure and binding prediction | A Comprehensive catalog of human DNaseI hypersensitive sites | U54 | UNIVERSITY OF WASHINGTON | 2011 |
| Regulatory genetics | Computational methods for modeling lineage-specific gene regulation | R01 | STANFORD UNIVERSITY | 2016 |
| Regulatory genetics | Computational Inference of Regulatory Network Dynamics on Cell Lineages | R01 | UNIVERSITY OF WISCONSIN-MADISON | 2019 |
| Regulatory genetics | Application of MAGNet Tools to Gene Regulatory Networks in Cancer | R01 | SLOAN-KETTERING INST CAN RESEARCH | 2008 |
| Regulatory genetics | Learning Regulatory Drivers of Chromatin and Expression Dynamics during Nuclear Reprogramming | R01 | STANFORD UNIVERSITY | 2019 |

© 2022 Eweje FR et al. *JAMA Network Open.*

| | | | | |
|---|---|---|---|---|
| Regulatory genetics | Global Discovery and Validation of Functional Regulatory Elements | R00 | UNIVERSITY OF MICHIGAN AT ANN ARBOR | 2017 |
| RNA analysis | Programmable RNA-targeting CRISPR-Cas tools to study RNA biology | R35 | UNIVERSITY OF ROCHESTER | 2020 |
| RNA analysis | Computational genome-wide RNA profiling using next-generation sequencing | R01 | UNIVERSITY OF PENNSYLVANIA | 2016 |
| RNA analysis | RNA STRUCTURE DETERMINATION WITH COMPARATIVE METHODS | R01 | UNIVERSITY OF COLORADO AT BOULDER | 1995 |
| RNA analysis | Discovery of Novel RNA Genes in Genomic DNA Sequences | R01 | UNIVERSITY OF CALIF-LAWRENC BERKELEY LAB | 2005 |
| RNA analysis | A Unified Nanopore Platform for Direct Sequencing of Individual Full Length RNA Strands Bearing Modified Nucleotides | R01 | UNIVERSITY OF CALIFORNIA SANTA CRUZ | 2020 |
| Schizophrenia | Genetic contributions to deficits in adaptive function in schizophrenia | K01 | MASSACHUSETTS GENERAL HOSPITAL | 2013 |
| Schizophrenia | Spatiotemporal imaging of language in schizophrenia | R01 | MASSACHUSETTS GENERAL HOSPITAL | 2009 |
| Schizophrenia | Brain abnormality in unaffected family members of schizophrenic patients | R03 | UNIVERSITY OF PENNSYLVANIA | 2008 |
| Schizophrenia | Application of Advanced Quantitative Methods to Schizophrenia Research in Macedonia | R56 | NEW YORK STATE PSYCHIATRIC INSTITUTE | 2019 |
| Schizophrenia | Pathway(s) From Genes to Functional Deficits of Schizophrenia Patients | R01 | UNIVERSITY OF CALIFORNIA, SAN DIEGO | 2010 |
| Sleep | Phylogeny of Sleep | R01 | BOSTON UNIVERSITY MEDICAL CAMPUS | 2007 |
| Sleep | Administrative Supplement for Sleep and Cardiometabolic Health Disparities at the US/Mexico Border: The Nogales Cardiometabolic Health and Sleep (NoCHeS) Study | R01 | UNIVERSITY OF ARIZONA | 2019 |
| Sleep | Sleep and Cardiometabolic Health Disparities at the US/Mexico Border: The Nogales Cardiometabolic Health and Sleep (NoCHeS) Study | R01 | UNIVERSITY OF ARIZONA | 2020 |
| Sleep | Cardiovascular implications of sleep characteristics using real-world objective sleep data | R21 | UNIVERSITY OF WASHINGTON | 2020 |
| Sleep | Genetic Analysis of Sleep Disorders in Zebrafish | R00 | CALIFORNIA INSTITUTE OF TECHNOLOGY | 2009 |
| Small molecule interactions | Ab-Initio Geometry Optimization of Large Molecules | R43 | Q-CHEM, INC. | 2003 |
| Small molecule interactions | Machine Learning in Chemistry and Biology | R01 | UNIVERSITY OF CALIFORNIA, SAN FRANCISCO | 2009 |
| Small molecule interactions | Reconstruction of heterogeneous and small macromolecules by cyro-EM | R01 | PRINCETON UNIVERSITY | 2020 |
| Small molecule interactions | Systematic Discovery of Bioactivation-Associated Structural Alerts | R01 | WASHINGTON UNIVERSITY | 2020 |
| Small molecule interactions | Binding-Site Modeling with Multiple-Instance Machine-Learning | R01 | UNIVERSITY OF CALIFORNIA, SAN FRANCISCO | 2016 |
| Social media and social behavior | Delineating proactive social behaviors in dynamic and multidimensional social space | R21 | ICAHN SCHOOL OF MEDICINE AT MOUNT SINAI | 2020 |
| Social media and social behavior | Dynamics of Large-Scale Networks During Emotional and Social Processing | R01 | UNIV OF MARYLAND, COLLEGE PARK | 2020 |
| Social media and social | Neural Circuit Mechanisms of Social Homeostasis in Individuals | DP1 | SALK INSTITUTE FOR BIOLOGICAL | 2019 |

© 2022 Eweje FR et al. *JAMA Network Open.*

| | | | | |
|---|---|---|---|---|
| behavior | and Supraorganismal Social Groups | | STUDIES | |
| Social media and social behavior | Studying how the hippocampal-prefrontal-hypothalamic circuit encodes social dominance | K99 | SALK INSTITUTE FOR BIOLOGICAL STUDIES | 2020 |
| Social media and social behavior | Supplementing Survey-Based Analyses of Group Vaccination Narratives and Behaviors Using Social Media | R01 | GEORGE WASHINGTON UNIVERSITY | 2019 |
| Speech | Speech Prosody and Articulatory Dynamics in Spoken Language | R01 | UNIVERSITY OF SOUTHERN CALIFORNIA | 2016 |
| Speech | Perception of dysarthric speech: An objective model of dysarthric speech evaluation with actionable outcomes | R01 | ARIZONA STATE UNIVERSITY-TEMPE CAMPUS | 2020 |
| Speech | The role of amplitude modulation in perceiving speech and music | F32 | NEW YORK UNIVERSITY | 2019 |
| Speech | Objectively Quantifying Speech Outcomes of Children with Cleft Palate | R21 | ARIZONA STATE UNIVERSITY-TEMPE CAMPUS | 2019 |
| Speech | Speech segregation to improve intelligility of reverberant-noisy speech | R01 | OHIO STATE UNIVERSITY | 2020 |
| Stroke | Biomarker panel to differentiate stroke from stroke mimic | R43 | PREDICTION SCIENCES, LLC | 2008 |
| Stroke | Fast and Easy Assessment of Stroke | R43 | ALVA HEALTH, INC. | 2019 |
| Stroke | Sleep apnea after stroke: Implications for screening and treatment | R01 | UNIVERSITY OF MICHIGAN AT ANN ARBOR | 2019 |
| Stroke | Whole Transcriptome Studies of Patients with Transient Ischemic Attacks (TIAs) | R01 | UNIVERSITY OF CALIFORNIA AT DAVIS | 2019 |
| Stroke | MEDIC ONE STROKE SCALE | K23 | UNIVERSITY OF WASHINGTON | 2003 |
| Student training and education | Initiative for Maximizing Student Development | R25 | UNIVERSITY OF MIAMI CORAL GABLES | 2007 |
| Student training and education | MUSC Minority Student Development Program | R25 | MEDICAL UNIVERSITY OF SOUTH CAROLINA | 2008 |
| Student training and education | Summer Institute in Geriatric Medicine | R13 | BOSTON UNIVERSITY MEDICAL CAMPUS | 2015 |
| Student training and education | Building a classroom game economy to improve mathematical reasoning and prepare K-5 students for success in STEM learning | R44 | TEACHLEY, LLC | 2020 |
| Student training and education | IPREP: IUPUI Graduate Preparation for the Biomedical and Behavioral Sciences. | R25 | INDIANA UNIV-PURDUE UNIV AT INDIANAPOLIS | 2020 |
| Suicidality | Predicting Self-Harm, Suicide Attempt, and Suicidal Death using Longitudinal EHR, Claims and Mortality Data | R01 | WEILL MEDICAL COLL OF CORNELL UNIV | 2020 |
| Suicidality | Predictive modeling: the role of opioid use in suicide risk | R01 | KAISER FOUNDATION RESEARCH INSTITUTE | 2020 |
| Suicidality | Digital Monitoring of Agitation for Short-Term Suicide Risk Prediction | K23 | MASSACHUSETTS GENERAL HOSPITAL | 2020 |
| Suicidality | Using multimodal imaging and the RDoC framework to predict risk factors for suicide attempt | R03 | YALE UNIVERSITY | 2020 |
| Suicidality | Statistical Methods for Predicting Suicide Attempt | K25 | COLUMBIA UNIVERSITY HEALTH SCIENCES | 2009 |
| Surgical planning | Outcome-Driven Approach to Minimize the Risks of Facial Distortion Following CMF Surgery | R01 | METHODIST HOSPITAL RESEARCH INSTITUTE | 2020 |
| Surgical planning | 3D Telestration for Robotically Assisted Surgery | R41 | INTUITIVE SURGICAL, INC. | 2004 |

© 2022 Eweje FR et al. *JAMA Network Open.*

| | | | | |
|---|---|---|---|---|
| Surgical planning | Quantifying the Metrics of Surgical Mastery: An Exploration in Data Science | R01 | STANFORD UNIVERSITY | 2020 |
| Surgical planning | Real-time non-intrusive workload monitoring-Integration of human factors in surgery training and assessment | R21 | PURDUE UNIVERSITY | 2020 |
| Surgical planning | Intelligent Virtual Reality Curriculum for Personalized Surgical Training | R43 | OSSO VR, INC. | 2019 |
| Text mining | Beyond Abstracts:  Issues in Mining Full Texts | R01 | UNIVERSITY OF COLORADO DENVER | 2008 |
| Text mining | Linking Text Mining and Data Mining for Biomedical Knowledge Discovery | G08 | UNIVERSITY OF DELAWARE | 2012 |
| Text mining | Evidence-based Strategy and Tool to Simplify Text for Patients and Consumers | R01 | UNIVERSITY OF ARIZONA | 2018 |
| Text mining | Advancing Literature Mining through Image Processing and Analysis | R01 | YALE UNIVERSITY | 2011 |
| Text mining | Incorporating Image-based Features into Biomedical Document Classification | R01 | UNIVERSITY OF DELAWARE | 2020 |
| Trauma | Measuring post-arrest neurologic injury via nanofluidic assay of brain-derived exosomal RNA | R21 | UNIVERSITY OF PENNSYLVANIA | 2019 |
| Trauma | NUTRIENT ESSENTIAL FATTY ACIDS & GASTRIC MUCOSAL INJURY | R01 | UNIVERSITY OF CALIFORNIA IRVINE | 1986 |
| Trauma | Leveraging Artificial-Intelligence to Profile and Enhance Phenotypic Plasticity for Second Injury Prevention: An Innovative Precision Medicine Platform to Revolutionize Injury Care | R21 | UNIV OF NORTH CAROLINA CHAPEL HILL | 2020 |
| Trauma | A nanomagnetic platform technology to characterize traumatic brain injury using brain derived extracellular vesicles | R33 | UNIVERSITY OF PENNSYLVANIA | 2020 |
| Trauma | Machine learning to inform health services and policy for traumatic brain injury | R01 | UNIVERSITY OF TORONTO | 2020 |
| Unspecified classification models | COMPONENT BASED TOOLS FOR CONNECTIONIST CLASSIFICATION | R01 | BRIGHAM AND WOMEN'S HOSPITAL | 1999 |
| Unspecified classification models | WAVELET-BASED AUTOMATED CHROMOSOME IDENTIFICATION | R44 | ADVANCED DIGITAL IMAGING RESEARCH, LLC | 2000 |
| Unspecified classification models | CLASSIFICATION OF NURSING-SENSITIVE PATIENT OUTCOMES | R01 | UNIVERSITY OF IOWA | 1997 |
| Unspecified classification models | WAVELET BASED AUTOMATED CHROMOSOME IDENTIFICATION | R43 | PERCEPTIVE SCIENTIFIC INSTRUMENTS, INC. | 1997 |
| Unspecified classification models | Position Sensitive P-Mer Frequency Clustering with Applications to Classification | R21 | SOUTHERN METHODIST UNIVERSITY | 2012 |
| Visual impairment | Benefit Assessment Tools for Substitute Prosthetic Regenerative and Ultra-Low Vision | R01 | JOHNS HOPKINS UNIVERSITY | 2020 |
| Visual impairment | Mid-Level Vision Systems for Low Vision | R01 | UNIVERSITY OF SOUTHERN CALIFORNIA | 2011 |
| Visual impairment | Addressing Low Vision due to Severe Peripheral Field Loss: Development and Validation of a Patient-Centered Outcome Measure | K23 | UNIVERSITY OF MICHIGAN AT ANN ARBOR | 2020 |
| Visual impairment | A software tool for objective identification of concussion-related vision disorders using a novel eye-tracking device | R41 | OCULOGICA, INC. | 2018 |
| Visual impairment | NRI: An Egocentric Computer Vision based Active Learning Co-Robot Wheelchair | R01 | STEVENS INSTITUTE OF TECHNOLOGY | 2017 |
| Visual processing | Towards cortical visual prosthetics | R21 | BOSTON CHILDREN'S HOSPITAL | 2010 |

© 2022 Eweje FR et al. *JAMA Network Open.*

| | | | | |
|---|---|---|---|---|
| Visual processing | Cue Reliability and Depth Calibration During Space Perception | R01 | STATE COLLEGE OF OPTOMETRY | 2011 |
| Visual processing | Learning and updating internal visual models | R01 | ALBERT EINSTEIN COLLEGE OF MEDICINE, INC | 2017 |
| Visual processing | Representation of information across the human visual cortex | R01 | UNIVERSITY OF CALIFORNIA BERKELEY | 2018 |
| Visual processing | Improving the quality of visual developmental EEG data with eye tracking | R03 | UNIVERSITY OF TEXAS DALLAS | 2010 |
| Wearable devices and mobile technology | Smartband/smartphone-based automatic smoking detection and real time mindfulness intervention | R34 | YALE UNIVERSITY | 2020 |
| Wearable devices and mobile technology | Developing Objective Assessment of Physical Activity and Sedentary Behavior for Adults with Down Syndrome | R15 | MISSISSIPPI STATE UNIVERSITY | 2019 |
| Wearable devices and mobile technology | An Intelligent Physical Activity Monitor | R43 | CREARE, INC. | 2006 |
| Wearable devices and mobile technology | Promoting Physical Activity in Latinas via Interactive Web-based Technology | R01 | UNIVERSITY OF CALIFORNIA, SAN DIEGO | 2015 |
| Wearable devices and mobile technology | A cutting edge approach to assessing physical activities occurring on sidewalks/streets | R21 | UNIVERSITY OF DELAWARE | 2019 |

© 2022 Eweje FR et al. *JAMA Network Open.*

**eTable 7.** National Institutes of Health funding for artificial intelligence by funding mechanism

| Mechanism | Number of awards (% of total number) | Value of awards (% of total value) |
|---|---|---|
| R01 | 7983 (48.0%) | $3,423,947,336 (47.7%) |
| R21 | 1137 (6.8%) | $239,977,819 (3.3%) |
| U01 | 800 (4.8%) | $579,492,289 (8.1%) |
| R44 | 692 (4.2%) | $336,221,031 (4.7%) |
| R43 | 683 (4.1%) | $108,573,518 (1.5%) |
| K01 | 388 (2.3%) | $56,369,585 (0.8%) |
| R03 | 338 (2.0%) | $31,824,822 (0.4%) |
| F31 | 304 (1.8%) | $10,713,644 (0.1%) |
| K23 | 303 (1.8%) | $52,314,972 (0.7%) |
| F32 | 244 (1.5%) | $12,498,483 (0.2%) |
| K99 | 233 (1.4%) | $24,872,177 (0.3%) |
| U24 | 198 (1.2%) | $280,855,683 (3.9%) |
| K08 | 191 (1.1%) | $30,773,785 (0.4%) |
| R29 | 174 (1.0%) | $17,036,001 (0.2%) |
| R00 | 145 (0.9%) | $34,140,613 (0.5%) |
| R35 | 145 (0.9%) | $58,915,647 (0.8%) |
| U54 | 138 (0.8%) | $175,716,122 (2.4%) |
| K25 | 124 (0.7%) | $17,269,773 (0.2%) |

© 2022 Eweje FR et al. *JAMA Network Open.*

| | | |
|---|---|---|
| R25 | 112 (0.7%) | $21,175,028 (0.3%) |
| P41 | 107 (0.6%) | $110,865,137 (1.5%) |
| R41 | 102 (0.6%) | $20,396,225 (0.3%) |
| R24 | 101 (0.6%) | $77,168,221 (1.1%) |
| R56 | 97 (0.6%) | $57,798,150 (0.8%) |
| F30 | 96 (0.6%) | $4,012,918 (0.1%) |
| R33 | 93 (0.6%) | $39,243,519 (0.5%) |
| N01 | 88 (0.5%) | $80,715,372 (1.1%) |
| P50 | 85 (0.5%) | $151,986,482 (2.1%) |
| R13 | 85 (0.5%) | $1,717,257 (0.0%) |
| R42 | 68 (0.4%) | $39,316,463 (0.5%) |
| K24 | 66 (0.4%) | $11,056,747 (0.2%) |
| U41 | 65 (0.4%) | $141,444,144 (2.0%) |
| P01 | 62 (0.4%) | $87,998,207 (1.2%) |
| U19 | 59 (0.4%) | $150,388,741 (2.1%) |
| K02 | 58 (0.3%) | $7,779,101 (0.1%) |
| K07 | 56 (0.3%) | $7,400,376 (0.1%) |
| P30 | 56 (0.3%) | $46,616,433 (0.6%) |
| R15 | 50 (0.3%) | $17,920,677 (0.2%) |
| RC1 | 48 (0.3%) | $23,291,530 (0.3%) |

© 2022 Eweje FR et al. *JAMA Network Open.*

| | | |
|---|---|---|
| RF1 | 48 (0.3%) | $93,352,304 (1.3%) |
| K22 | 39 (0.2%) | $6,061,523 (0.1%) |
| R34 | 39 (0.2%) | $9,134,051 (0.1%) |
| R37 | 39 (0.2%) | $14,125,679 (0.2%) |
| G08 | 38 (0.2%) | $8,234,969 (0.1%) |
| UH2 | 32 (0.2%) | $12,706,050 (0.2%) |
| UH3 | 32 (0.2%) | $24,538,922 (0.3%) |
| DP2 | 31 (0.2%) | $56,396,654 (0.8%) |
| N43 | 31 (0.2%) | $8,758,866 (0.1%) |
| RC2 | 30 (0.2%) | $47,746,112 (0.7%) |
| R61 | 28 (0.2%) | $37,939,177 (0.5%) |
| R18 | 24 (0.1%) | $9,205,118 (0.1%) |
| UG3 | 23 (0.1%) | $10,333,760 (0.1%) |
| DP1 | 22 (0.1%) | $19,838,149 (0.3%) |
| P20 | 22 (0.1%) | $17,591,775 (0.2%) |
| DP5 | 21 (0.1%) | $7,630,616 (0.1%) |
| S10 | 20 (0.1%) | $21,646,130 (0.3%) |
| N02 | 19 (0.1%) | $7,311,377 (0.1%) |
| K04 | 17 (0.1%) | $1,023,966 (0.0%) |
| N03 | 16 (0.1%) | $7,576,588 (0.1%) |

© 2022 Eweje FR et al. *JAMA Network Open.*

| | | |
|---|---|---|
| SC1 | 15 (0.1%) | $4,532,625 (0.1%) |
| UG1 | 15 (0.1%) | $28,172,619 (0.4%) |
| UL1 | 15 (0.1%) | $19,792,963 (0.3%) |
| OT2 | 14 (0.1%) | $12,980,385 (0.2%) |
| U2C | 13 (0.1%) | $22,804,984 (0.3%) |
| F38 | 12 (0.1%) | $745,217 (0.0%) |
| U18 | 12 (0.1%) | $6,419,748 (0.1%) |
| Y01 | 11 (0.1%) | $3,943,597 (0.1%) |
| F99 | 10 (0.1%) | $349,828 (0.0%) |
| OT3 | 10 (0.1%) | $7,774,024 (0.1%) |
| R90 | 10 (0.1%) | $2,398,177 (0.0%) |
| SC2 | 10 (0.1%) | $1,406,895 (0.0%) |
| F37 | 9 (0.1%) | $398,860 (0.0%) |
| K00 | 9 (0.1%) | $652,810 (0.0%) |
| R36 | 9 (0.1%) | $412,491 (0.0%) |
| K05 | 8 (0.0%) | $1,370,394 (0.0%) |
| SC3 | 8 (0.0%) | $592,686 (0.0%) |
| K12 | 7 (0.0%) | $2,892,835 (0.0%) |
| R23 | 7 (0.0%) | $359,827 (0.0%) |
| UM1 | 7 (0.0%) | $9,187,868 (0.1%) |

© 2022 Eweje FR et al. *JAMA Network Open.*

| | | |
|---|---|---|
| U44 | 6 (0.0%) | $1,967,425 (0.0%) |
| KL2 | 5 (0.0%) | $1,972,252 (0.0%) |
| RM1 | 5 (0.0%) | $7,112,351 (0.1%) |
| K11 | 4 (0.0%) | $301,246 (0.0%) |
| K18 | 4 (0.0%) | $579,270 (0.0%) |
| K76 | 4 (0.0%) | $921,902 (0.0%) |
| N44 | 4 (0.0%) | $2,739,641 (0.0%) |
| G13 | 3 (0.0%) | $224,846 (0.0%) |
| K43 | 3 (0.0%) | $185,322 (0.0%) |
| P60 | 3 (0.0%) | $5,428,065 (0.1%) |
| R55 | 3 (0.0%) | $200,000 (0.0%) |
| S06 | 3 (0.0%) | $343,511 (0.0%) |
| S07 | 3 (0.0%) | $233,689 (0.0%) |
| U10 | 3 (0.0%) | $668,360 (0.0%) |
| U13 | 3 (0.0%) | $404,893 (0.0%) |
| DP3 | 2 (0.0%) | $5,024,500 (0.1%) |
| F33 | 2 (0.0%) | $73,995 (0.0%) |
| P2C | 2 (0.0%) | $1,833,600 (0.0%) |
| R50 | 2 (0.0%) | $253,333 (0.0%) |
| U34 | 2 (0.0%) | $718,747 (0.0%) |

© 2022 Eweje FR et al. *JAMA Network Open.*

| | | |
|---|---|---|
| D43 | 1 (0.0%) | $73,250 (0.0%) |
| G20 | 1 (0.0%) | $500,000 (0.0%) |
| P42 | 1 (0.0%) | $2,414,074 (0.0%) |
| RL1 | 1 (0.0%) | $303,654 (0.0%) |
| SB1 | 1 (0.0%) | $300,000 (0.0%) |
| UC2 | 1 (0.0%) | $1,957,980 (0.0%) |
| UF1 | 1 (0.0%) | $2,200,000 (0.0%) |

© 2022 Eweje FR et al. *JAMA Network Open.*

**eTable 8.** Funding mechanism frequencies among National Institutes of Health-funded artificial intelligence applications in biomedical research

| Application Category | Application | No. of R01 (% in application) | No. of U01 (% in application) | No. of R44 (% in application) | No. of R21 (% in application) | Total number of awards |
|---|---|---|---|---|---|---|
| Biochemical analysis | Cell signaling pathways | 74 (63%) | 11 (9%) | 3 (3%) | 11 (9%) | 117 |
| | Drug discovery | 67 (43%) | 0 (0%) | 8 (5%) | 13 (8%) | 156 |
| | Mass spectroscopy | 104 (72%) | 3 (2%) | 3 (2%) | 8 (6%) | 145 |
| | Other chemical compound characterization | 65 (41%) | 10 (6%) | 14 (9%) | 12 (8%) | 159 |
| | Protein structure and binding prediction | 69 (53%) | 10 (8%) | 7 (5%) | 6 (5%) | 130 |
| | Small molecule interactions | 41 (51%) | 2 (2%) | 3 (4%) | 4 (5%) | 81 |
| | **Total** | **420 (53%)** | **36 (5%)** | **38 (5%)** | **54 (7%)** | **788** |
| Cancer | Breast cancer | 132 (44%) | 26 (9%) | 8 (3%) | 25 (8%) | 297 |
| | Other cancer | 156 (42%) | 48 (13%) | 9 (2%) | 24 (6%) | 375 |
| | Prostate cancer | 61 (48%) | 10 (8%) | 2 (2%) | 22 (17%) | 127 |
| | **Total** | **349 (44%)** | **36 (5%)** | **38 (5%)** | **54 (7%)** | **799** |
| Cardiovascular | Cardiovascular disease | 99 (53%) | 1 (1%) | 6 (3%) | 11 (6%) | 186 |
| | **Total** | **99 (53%)** | **36 (5%)** | **38 (5%)** | **54 (7%)** | **186** |
| Data types | Big data | 35 (26%) | 5 (4%) | 5 (4%) | 4 (3%) | 135 |
| | Motion tracking and artifact reduction | 76 (51%) | 2 (1%) | 10 (7%) | 14 (9%) | 150 |
| | Text mining | 131 (60%) | 9 (4%) | 7 (3%) | 9 (4%) | 220 |
| | Wearable devices and mobile technology | 93 (48%) | 14 (7%) | 8 (4%) | 23 (12%) | 193 |

© 2022 Eweje FR et al. *JAMA Network Open.*

| | | | | | | |
|---|---|---|---|---|---|---|
| | **Total** | **335 (48%)** | **36 (5%)** | **38 (5%)** | **54 (7%)** | **698** |
| Electronic health record | Electronic health record | 139 (47%) | 28 (9%) | 2 (1%) | 22 (7%) | 296 |
| | **Total** | **139 (47%)** | **36 (5%)** | **38 (5%)** | **54 (7%)** | **296** |
| Endocrine | Diabetes | 47 (41%) | 13 (11%) | 2 (2%) | 10 (9%) | 114 |
| | Metabolic syndrome and metabolic processes | 63 (64%) | 8 (8%) | 2 (2%) | 3 (3%) | 99 |
| | **Total** | **110 (52%)** | **36 (5%)** | **38 (5%)** | **54 (7%)** | **213** |
| Environmental health | Environmental health | 105 (52%) | 4 (2%) | 1 (0%) | 19 (9%) | 203 |
| | **Total** | **105 (52%)** | **36 (5%)** | **38 (5%)** | **54 (7%)** | **203** |
| Genetics | Clinically significant genetic variation | 127 (54%) | 37 (16%) | 1 (0%) | 4 (2%) | 236 |
| | Familial genetics | 61 (47%) | 28 (22%) | 3 (2%) | 0 (0%) | 129 |
| | Functional mutations | 89 (55%) | 16 (10%) | 3 (2%) | 9 (6%) | 162 |
| | Gene mapping | 101 (61%) | 6 (4%) | 2 (1%) | 8 (5%) | 166 |
| | Molecular genetics | 120 (49%) | 12 (5%) | 16 (7%) | 15 (6%) | 246 |
| | Mouse modeling | 85 (51%) | 10 (6%) | 5 (3%) | 19 (11%) | 167 |
| | Population genetics | 89 (68%) | 9 (7%) | 5 (4%) | 6 (5%) | 130 |
| | Regulatory genetics | 184 (66%) | 20 (7%) | 4 (1%) | 4 (1%) | 280 |
| | RNA analysis | 57 (49%) | 0 (0%) | 6 (5%) | 9 (8%) | 116 |
| | **Total** | **913 (56%)** | **36 (5%)** | **38 (5%)** | **54 (7%)** | **1632** |
| Hepatic | Liver disease | 65 (53%) | 24 (20%) | 0 (0%) | 9 (7%) | 123 |
| | Total | 65 (53%) | 36 (5%) | 38 (5%) | 54 (7%) | 123 |

© 2022 Eweje FR et al. *JAMA Network Open.*

| | | | | | | |
|---|---|---|---|---|---|---|
| Infectious disease/Immunologic | HIV | 102 (42%) | 13 (5%) | 4 (2%) | 27 (11%) | 243 |
| | Immunology | 67 (39%) | 12 (7%) | 4 (2%) | 13 (8%) | 170 |
| | Other infectious disease | 83 (34%) | 20 (8%) | 2 (1%) | 35 (14%) | 242 |
| | **Total** | **252 (38%)** | **36 (5%)** | **38 (5%)** | **54 (7%)** | **655** |
| Injuries/trauma | Trauma | 75 (47%) | 8 (5%) | 3 (2%) | 25 (16%) | 159 |
| | **Total** | **75 (47%)** | **36 (5%)** | **38 (5%)** | **54 (7%)** | **159** |
| Knowledge frameworks | Centers for translational and computational research | 17 (12%) | 13 (9%) | 1 (1%) | 2 (1%) | 144 |
| | Intelligent search engines and data visualization | 28 (37%) | 3 (4%) | 1 (1%) | 1 (1%) | 76 |
| | Knowledge bases | 61 (35%) | 12 (7%) | 2 (1%) | 4 (2%) | 176 |
| | Knowledge representation and reasoning | 80 (66%) | 0 (0%) | 4 (3%) | 5 (4%) | 122 |
| | Literature review | 34 (45%) | 0 (0%) | 0 (0%) | 5 (7%) | 76 |
| | Ontology generation | 59 (60%) | 4 (4%) | 5 (5%) | 2 (2%) | 99 |
| | **Total** | **279 (40%)** | **36 (5%)** | **38 (5%)** | **54 (7%)** | **693** |
| Language and communication | Interpersonal communication technologies | 49 (53%) | 5 (5%) | 4 (4%) | 5 (5%) | 92 |
| | Language development and reading comprehension | 159 (59%) | 14 (5%) | 10 (4%) | 21 (8%) | 271 |
| | Social media and social behavior | 121 (57%) | 6 (3%) | 5 (2%) | 18 (8%) | 212 |
| | Speech | 134 (64%) | 7 (3%) | 10 (5%) | 14 (7%) | 210 |
| | **Total** | **463 (59%)** | **36 (5%)** | **38 (5%)** | **54 (7%)** | **785** |
| Mental health | Adolescent psychiatry | 49 (32%) | 16 (10%) | 5 (3%) | 12 (8%) | 155 |
| | Alcohol use | 70 (32%) | 53 (24%) | 0 (0%) | 15 (7%) | 218 |

© 2022 Eweje FR et al. *JAMA Network Open.*

| | | | | | | |
|---|---|---|---|---|---|---|
| | Autism spectrum disorder | 92 (48%) | 3 (2%) | 11 (6%) | 17 (9%) | 192 |
| | Depression | 43 (41%) | 8 (8%) | 6 (6%) | 9 (9%) | 104 |
| | Other child development | 75 (46%) | 7 (4%) | 1 (1%) | 9 (5%) | 164 |
| | Other mental health | 55 (37%) | 4 (3%) | 6 (4%) | 5 (3%) | 148 |
| | Pain | 97 (52%) | 0 (0%) | 4 (2%) | 17 (9%) | 188 |
| | Schizophrenia | 57 (50%) | 4 (4%) | 0 (0%) | 16 (14%) | 113 |
| | Suicidality | 42 (53%) | 3 (4%) | 3 (4%) | 5 (6%) | 79 |
| | **Total** | **580 (43%)** | **36 (5%)** | **38 (5%)** | **54 (7%)** | **1361** |
| Model types | Deep learning | 98 (44%) | 9 (4%) | 13 (6%) | 35 (16%) | 221 |
| | Natural language processing | 68 (56%) | 9 (7%) | 1 (1%) | 12 (10%) | 121 |
| | Unspecified classification models | 63 (48%) | 1 (1%) | 10 (8%) | 10 (8%) | 131 |
| | **Total** | **229 (48%)** | **36 (5%)** | **38 (5%)** | **54 (7%)** | **473** |
| Neurologic | Alzheimer's disease | 176 (55%) | 17 (5%) | 8 (2%) | 21 (7%) | 321 |
| | EEG | 43 (41%) | 4 (4%) | 20 (19%) | 7 (7%) | 104 |
| | Memory | 73 (49%) | 4 (3%) | 0 (0%) | 8 (5%) | 149 |
| | Motor function | 101 (53%) | 11 (6%) | 5 (3%) | 14 (7%) | 192 |
| | Neural circuits | 199 (55%) | 21 (6%) | 3 (1%) | 21 (6%) | 361 |
| | Other dementia | 71 (48%) | 0 (0%) | 19 (13%) | 8 (5%) | 148 |
| | Sleep | 37 (37%) | 0 (0%) | 10 (10%) | 11 (11%) | 101 |
| | Stroke | 89 (51%) | 0 (0%) | 0 (0%) | 11 (6%) | 176 |

© 2022 Eweje FR et al. *JAMA Network Open.*

| | | | | | | |
|---|---|---|---|---|---|---|
| | **Total** | **789 (51%)** | **36 (5%)** | **38 (5%)** | **54 (7%)** | **1552** |
| Population health | Population health screening | 62 (41%) | 2 (1%) | 12 (8%) | 18 (12%) | 151 |
| | Older adults | 94 (55%) | 0 (0%) | 1 (1%) | 9 (5%) | 171 |
| | Pediatrics | 26 (41%) | 4 (6%) | 2 (3%) | 9 (14%) | 63 |
| | **Total** | **182 (47%)** | **36 (5%)** | **38 (5%)** | **54 (7%)** | **385** |
| Patient safety | Adverse drug events/drug safety | 129 (49%) | 12 (5%) | 15 (6%) | 16 (6%) | 265 |
| | Other patient safety | 73 (63%) | 0 (0%) | 8 (7%) | 5 (4%) | 115 |
| | Surgical planning | 71 (52%) | 2 (1%) | 9 (7%) | 17 (12%) | 137 |
| | **Total** | **273 (53%)** | **36 (5%)** | **38 (5%)** | **54 (7%)** | **517** |
| Renal | Kidney disease | 58 (47%) | 0 (0%) | 4 (3%) | 13 (11%) | 123 |
| | **Total** | **58 (47%)** | **36 (5%)** | **38 (5%)** | **54 (7%)** | **123** |
| Respiratory | Asthma | 25 (27%) | 5 (5%) | 2 (2%) | 12 (13%) | 93 |
| | Lung cancer and COPD | 113 (52%) | 6 (3%) | 4 (2%) | 17 (8%) | 217 |
| | **Total** | **138 (45%)** | **36 (5%)** | **38 (5%)** | **54 (7%)** | **310** |
| Training and education | Student training and education | 29 (19%) | 0 (0%) | 17 (11%) | 0 (0%) | 149 |
| | **Total** | **29 (19%)** | **36 (5%)** | **38 (5%)** | **54 (7%)** | **149** |
| Vision | Object tracking and recognition | 75 (66%) | 0 (0%) | 2 (2%) | 6 (5%) | 113 |
| | Visual impairment | 37 (47%) | 0 (0%) | 2 (3%) | 7 (9%) | 78 |
| | Visual processing | 120 (71%) | 0 (0%) | 6 (4%) | 9 (5%) | 168 |
| | **Total** | **232 (65%)** | **36 (5%)** | **38 (5%)** | **54 (7%)** | **359** |

© 2022 Eweje FR et al. *JAMA Network Open.*

**eTable 9.** Comparison of National Institutes of Health funding mechanism frequency for the four most common funding mechanisms, by pairwise general application categories. Comparisons are conducted using exact binomial tests with post-hoc Bonferroni correction. Only comparisons that remained statistically significant after correction are included.

| Funding Mechanism | Application category 1 | Application category 2 | Funding mechanism frequency in category 1 | Funding mechanism frequency in category 2 | p-value (Bonferroni corrected) |
|---|---|---|---|---|---|
| R01 | Biochemical analysis | Cancer | 53.3% | 43.7% | 0.031 |
| R01 | Biochemical analysis | Infectious disease/Immunologic | 53.3% | 38.5% | <0.001 |
| R01 | Biochemical analysis | Knowledge frameworks | 53.3% | 40.3% | <0.001 |
| R01 | Biochemical analysis | Mental health | 53.3% | 42.6% | <0.001 |
| R01 | Biochemical analysis | Training and education | 53.3% | 19.5% | <0.001 |
| R01 | Cancer | Genetics | 43.7% | 55.9% | <0.001 |
| R01 | Cancer | Language and communication | 43.7% | 59.0% | <0.001 |
| R01 | Cancer | Training and education | 43.7% | 19.5% | <0.001 |
| R01 | Cancer | Vision | 43.7% | 64.6% | <0.001 |
| R01 | Cardiovascular | Training and education | 53.2% | 19.5% | <0.001 |
| R01 | Data types | Language and communication | 48.0% | 59.0% | 0.005 |
| R01 | Data types | Training and education | 48.0% | 19.5% | <0.001 |
| R01 | Data types | Vision | 48.0% | 64.6% | <0.001 |
| R01 | Electronic health record | Training and education | 47.0% | 19.5% | <0.001 |
| R01 | Electronic health record | Vision | 47.0% | 64.6% | 0.001 |
| R01 | Endocrine | Training and education | 51.6% | 19.5% | <0.001 |

© 2022 Eweje FR et al. *JAMA Network Open.*

| | | | | | |
|---|---|---|---|---|---|
| R01 | Environmental health | Training and education | 51.7% | 19.5% | <0.001 |
| R01 | Genetics | Infectious disease/Immunologic | 55.9% | 38.5% | <0.001 |
| R01 | Genetics | Knowledge frameworks | 55.9% | 40.3% | <0.001 |
| R01 | Genetics | Mental health | 55.9% | 42.6% | <0.001 |
| R01 | Genetics | Training and education | 55.9% | 19.5% | <0.001 |
| R01 | Hepatic | Training and education | 52.8% | 19.5% | <0.001 |
| R01 | Infectious disease/Immunologic | Language and communication | 38.5% | 59.0% | <0.001 |
| R01 | Infectious disease/Immunologic | Neurologic | 38.5% | 50.8% | <0.001 |
| R01 | Infectious disease/Immunologic | Patient safety | 38.5% | 52.8% | <0.001 |
| R01 | Infectious disease/Immunologic | Training and education | 38.5% | 19.5% | 0.002 |
| R01 | Infectious disease/Immunologic | Vision | 38.5% | 64.6% | <0.001 |
| R01 | Injuries/trauma | Training and education | 47.2% | 19.5% | <0.001 |
| R01 | Knowledge frameworks | Language and communication | 40.3% | 59.0% | <0.001 |
| R01 | Knowledge frameworks | Neurologic | 40.3% | 50.8% | <0.001 |
| R01 | Knowledge frameworks | Patient safety | 40.3% | 52.8% | 0.004 |
| R01 | Knowledge frameworks | Training and education | 40.3% | 19.5% | <0.001 |
| R01 | Knowledge frameworks | Vision | 40.3% | 64.6% | <0.001 |
| R01 | Language and communication | Mental health | 59.0% | 42.6% | <0.001 |
| R01 | Language and communication | Population health | 59.0% | 47.3% | 0.040 |
| R01 | Language and communication | Respiratory | 59.0% | 44.5% | 0.004 |

© 2022 Eweje FR et al. *JAMA Network Open.*

| | | | | | |
|---|---|---|---|---|---|
| R01 | Language and communication | Training and education | 59.0% | 19.5% | <0.001 |
| R01 | Mental health | Neurologic | 42.6% | 50.8% | 0.002 |
| R01 | Mental health | Patient safety | 42.6% | 52.8% | 0.018 |
| R01 | Mental health | Training and education | 42.6% | 19.5% | <0.001 |
| R01 | Mental health | Vision | 42.6% | 64.6% | <0.001 |
| R01 | Model types | Training and education | 48.4% | 19.5% | <0.001 |
| R01 | Model types | Vision | 48.4% | 64.6% | <0.001 |
| R01 | Neurologic | Training and education | 50.8% | 19.5% | <0.001 |
| R01 | Neurologic | Vision | 50.8% | 64.6% | <0.001 |
| R01 | Patient safety | Training and education | 52.8% | 19.5% | <0.001 |
| R01 | Population health | Training and education | 47.3% | 19.5% | <0.001 |
| R01 | Population health | Vision | 47.3% | 64.6% | <0.001 |
| R01 | Renal | Training and education | 47.2% | 19.5% | <0.001 |
| R01 | Respiratory | Training and education | 44.5% | 19.5% | <0.001 |
| R01 | Respiratory | Vision | 44.5% | 64.6% | <0.001 |
| R01 | Training and education | Vision | 19.5% | 64.6% | <0.001 |
| R21 | Biochemical analysis | Training and education | 6.9% | 0.0% | 0.033 |
| R21 | Cancer | Genetics | 8.9% | 4.5% | 0.008 |
| R21 | Cancer | Knowledge frameworks | 8.9% | 2.7% | <0.001 |
| R21 | Cancer | Training and education | 8.9% | 0.0% | 0.001 |

© 2022 Eweje FR et al. *JAMA Network Open.*

| | | | | | |
|---|---|---|---|---|---|
| R21 | Data types | Knowledge frameworks | 7.2% | 2.7% | 0.040 |
| R21 | Data types | Training and education | 7.2% | 0.0% | 0.016 |
| R21 | Electronic health record | Training and education | 7.4% | 0.0% | 0.027 |
| R21 | Environmental health | Knowledge frameworks | 9.4% | 2.7% | 0.044 |
| R21 | Environmental health | Training and education | 9.4% | 0.0% | 0.009 |
| R21 | Genetics | Infectious disease/Immunologic | 4.5% | 11.5% | <0.001 |
| R21 | Genetics | Injuries/trauma | 4.5% | 15.7% | <0.001 |
| R21 | Genetics | Model types | 4.5% | 12.1% | <0.001 |
| R21 | Infectious disease/Immunologic | Knowledge frameworks | 11.5% | 2.7% | <0.001 |
| R21 | Infectious disease/Immunologic | Neurologic | 11.5% | 6.5% | 0.033 |
| R21 | Infectious disease/Immunologic | Training and education | 11.5% | 0.0% | <0.001 |
| R21 | Injuries/trauma | Knowledge frameworks | 15.7% | 2.7% | <0.001 |
| R21 | Injuries/trauma | Neurologic | 15.7% | 6.5% | 0.036 |
| R21 | Injuries/trauma | Training and education | 15.7% | 0.0% | <0.001 |
| R21 | Knowledge frameworks | Language and communication | 2.7% | 7.4% | 0.012 |
| R21 | Knowledge frameworks | Mental health | 2.7% | 7.7% | <0.001 |
| R21 | Knowledge frameworks | Model types | 2.7% | 12.1% | <0.001 |
| R21 | Knowledge frameworks | Neurologic | 2.7% | 6.5% | 0.035 |
| R21 | Knowledge frameworks | Population health | 2.7% | 9.4% | 0.001 |
| R21 | Knowledge frameworks | Respiratory | 2.7% | 9.4% | 0.004 |

© 2022 Eweje FR et al. *JAMA Network Open.*

| | | | | | |
|---|---|---|---|---|---|
| R21 | Language and communication | Training and education | 7.4% | 0.0% | 0.012 |
| R21 | Mental health | Training and education | 7.7% | 0.0% | 0.005 |
| R21 | Model types | Neurologic | 12.1% | 6.5% | 0.041 |
| R21 | Model types | Training and education | 12.1% | 0.0% | <0.001 |
| R21 | Neurologic | Training and education | 6.5% | 0.0% | 0.037 |
| R21 | Patient safety | Training and education | 7.4% | 0.0% | 0.017 |
| R21 | Population health | Training and education | 9.4% | 0.0% | 0.002 |
| R21 | Renal | Training and education | 10.6% | 0.0% | 0.005 |
| R21 | Respiratory | Training and education | 9.4% | 0.0% | 0.003 |
| R44 | Cancer | Training and education | 2.4% | 11.4% | 0.001 |
| R44 | Electronic health record | Patient safety | 0.7% | 6.2% | 0.009 |
| R44 | Electronic health record | Training and education | 0.7% | 11.4% | <0.001 |
| R44 | Endocrine | Training and education | 1.9% | 11.4% | 0.039 |
| R44 | Environmental health | Training and education | 0.5% | 11.4% | <0.001 |
| R44 | Genetics | Training and education | 2.8% | 11.4% | 0.001 |
| R44 | Hepatic | Training and education | 0.0% | 11.4% | 0.009 |
| R44 | Infectious disease/Immunologic | Patient safety | 1.5% | 6.2% | 0.005 |
| R44 | Infectious disease/Immunologic | Training and education | 1.5% | 11.4% | <0.001 |
| R44 | Knowledge frameworks | Patient safety | 1.9% | 6.2% | 0.037 |
| R44 | Knowledge frameworks | Training and education | 1.9% | 11.4% | <0.001 |

© 2022 Eweje FR et al. *JAMA Network Open.*

| | | | | | |
|---|---|---|---|---|---|
| R44 | Mental health | Training and education | 2.6% | 11.4% | 0.001 |
| R44 | Respiratory | Training and education | 1.9% | 11.4% | 0.008 |
| U01 | Biochemical analysis | Cancer | 4.6% | 10.5% | 0.002 |
| U01 | Biochemical analysis | Hepatic | 4.6% | 19.5% | <0.001 |
| U01 | Biochemical analysis | Vision | 4.6% | 0.0% | <0.001 |
| U01 | Cancer | Cardiovascular | 10.5% | 0.5% | <0.001 |
| U01 | Cancer | Data types | 10.5% | 4.3% | 0.001 |
| U01 | Cancer | Environmental health | 10.5% | 2.0% | 0.005 |
| U01 | Cancer | Knowledge frameworks | 10.5% | 4.6% | 0.004 |
| U01 | Cancer | Language and communication | 10.5% | 4.1% | <0.001 |
| U01 | Cancer | Model types | 10.5% | 4.0% | 0.006 |
| U01 | Cancer | Neurologic | 10.5% | 3.7% | <0.001 |
| U01 | Cancer | Patient safety | 10.5% | 2.7% | <0.001 |
| U01 | Cancer | Population health | 10.5% | 1.6% | <0.001 |
| U01 | Cancer | Renal | 10.5% | 0.0% | 0.001 |
| U01 | Cancer | Respiratory | 10.5% | 3.5% | 0.025 |
| U01 | Cancer | Training and education | 10.5% | 0.0% | <0.001 |
| U01 | Cancer | Vision | 10.5% | 0.0% | <0.001 |
| U01 | Cardiovascular | Electronic health record | 0.5% | 9.5% | 0.003 |
| U01 | Cardiovascular | Endocrine | 0.5% | 9.9% | 0.005 |

© 2022 Eweje FR et al. *JAMA Network Open.*

| | | | | | |
|---|---|---|---|---|---|
| U01 | Cardiovascular | Genetics | 0.5% | 8.5% | 0.001 |
| U01 | Cardiovascular | Hepatic | 0.5% | 19.5% | <0.001 |
| U01 | Cardiovascular | Infectious disease/Immunologic | 0.5% | 6.9% | 0.039 |
| U01 | Cardiovascular | Mental health | 0.5% | 7.2% | 0.010 |
| U01 | Data types | Hepatic | 4.3% | 19.5% | <0.001 |
| U01 | Data types | Vision | 4.3% | 0.0% | 0.001 |
| U01 | Electronic health record | Neurologic | 9.5% | 3.7% | 0.023 |
| U01 | Electronic health record | Patient safety | 9.5% | 2.7% | 0.012 |
| U01 | Electronic health record | Population health | 9.5% | 1.6% | <0.001 |
| U01 | Electronic health record | Renal | 9.5% | 0.0% | 0.013 |
| U01 | Electronic health record | Training and education | 9.5% | 0.0% | 0.003 |
| U01 | Electronic health record | Vision | 9.5% | 0.0% | <0.001 |
| U01 | Endocrine | Patient safety | 9.9% | 2.7% | 0.038 |
| U01 | Endocrine | Population health | 9.9% | 1.6% | 0.002 |
| U01 | Endocrine | Renal | 9.9% | 0.0% | 0.016 |
| U01 | Endocrine | Training and education | 9.9% | 0.0% | 0.003 |
| U01 | Endocrine | Vision | 9.9% | 0.0% | <0.001 |
| U01 | Environmental health | Hepatic | 2.0% | 19.5% | <0.001 |
| U01 | Genetics | Language and communication | 8.5% | 4.1% | 0.013 |
| U01 | Genetics | Neurologic | 8.5% | 3.7% | <0.001 |

© 2022 Eweje FR et al. *JAMA Network Open.*

| | | | | | |
|---|---|---|---|---|---|
| U01 | Genetics | Patient safety | 8.5% | 2.7% | <0.001 |
| U01 | Genetics | Population health | 8.5% | 1.6% | <0.001 |
| U01 | Genetics | Renal | 8.5% | 0.0% | 0.016 |
| U01 | Genetics | Training and education | 8.5% | 0.0% | 0.002 |
| U01 | Genetics | Vision | 8.5% | 0.0% | <0.001 |
| U01 | Hepatic | Infectious disease/Immunologic | 19.5% | 6.9% | 0.009 |
| U01 | Hepatic | Knowledge frameworks | 19.5% | 4.6% | <0.001 |
| U01 | Hepatic | Language and communication | 19.5% | 4.1% | <0.001 |
| U01 | Hepatic | Mental health | 19.5% | 7.2% | 0.009 |
| U01 | Hepatic | Model types | 19.5% | 4.0% | <0.001 |
| U01 | Hepatic | Neurologic | 19.5% | 3.7% | <0.001 |
| U01 | Hepatic | Patient safety | 19.5% | 2.7% | <0.001 |
| U01 | Hepatic | Population health | 19.5% | 1.6% | <0.001 |
| U01 | Hepatic | Renal | 19.5% | 0.0% | <0.001 |
| U01 | Hepatic | Respiratory | 19.5% | 3.5% | <0.001 |
| U01 | Hepatic | Training and education | 19.5% | 0.0% | <0.001 |
| U01 | Hepatic | Vision | 19.5% | 0.0% | <0.001 |
| U01 | Infectious disease/Immunologic | Population health | 6.9% | 1.6% | 0.017 |
| U01 | Infectious disease/Immunologic | Training and education | 6.9% | 0.0% | 0.025 |
| U01 | Infectious disease/Immunologic | Vision | 6.9% | 0.0% | <0.001 |

© 2022 Eweje FR et al. *JAMA Network Open.*

| | | | | | |
|---|---|---|---|---|---|
| U01 | Injuries/trauma | Vision | 5.0% | 0.0% | 0.016 |
| U01 | Knowledge frameworks | Vision | 4.6% | 0.0% | <0.001 |
| U01 | Language and communication | Vision | 4.1% | 0.0% | 0.002 |
| U01 | Mental health | Neurologic | 7.2% | 3.7% | 0.007 |
| U01 | Mental health | Patient safety | 7.2% | 2.7% | 0.027 |
| U01 | Mental health | Population health | 7.2% | 1.6% | 0.002 |
| U01 | Mental health | Training and education | 7.2% | 0.0% | 0.014 |
| U01 | Mental health | Vision | 7.2% | 0.0% | <0.001 |
| U01 | Model types | Vision | 4.0% | 0.0% | 0.005 |
| U01 | Neurologic | Vision | 3.7% | 0.0% | 0.002 |
| U01 | Respiratory | Vision | 3.5% | 0.0% | 0.044 |

© 2022 Eweje FR et al. *JAMA Network Open.*
